# Supplementary material for: Socioeconomic position indicators and risk of alcohol-related medical conditions: A national cohort study from Sweden
Source: PLoS Med. 2024 Mar 19;21(3):e1004359. doi: 10.1371/journal.pmed.1004359 (PMC10950249; doi:10.1371/journal.pmed.1004359)
Supplement: S1 Methods — (DOCX) [file pmed.1004359.s001.docx]

**S1 Methods**

*Registry resources*

The following sources were used to create our dataset: Total Population Register, containing information about year of birth, sex, family and marital status; Multi-Generation Register, linking individuals born after 1932 to their parents; The longitudinal integrated database for health insurance and labour market studies (LISA) with yearly information on income and education from 2000 to 2018, the Hospital Discharge Register, containing hospitalizations for Swedish inhabitants from 1964-2018; Prescribed Drug Register, containing all prescriptions in Sweden picked up by patients from July 2005 to 2018; Outpatient Care Register, containing information from all outpatient clinics from 2001 to 2018; and regional Primary Health Care Registers data from Blekinge (2009 - 2016), Dalarna (2005 - 2013), Värmland (2005 - 2015), Kalmar Län (2007 - 2016), Sörmland (1992 - 2017), Uppsala Län (2005 - 2015), Västernorrland (2008 - 2015) Norrbotten Län (2001 - 2014), Gävleborg (2010 - 2017), Gotland (2011 – 2018), Halland (2007 - 2014), Jönköpings Län (2008 - 2014), Kronoberg (2006 - 2016), Skåne (1989 - 2018), Västerbotten (1992- 2018), Östergötland (1990 - 2014), Stockholms Län (2003 - 2016), and Västra Götaland (2000 - 2013). The time-periods varies due to the regions different timing of digitalizing of the patient records. In addition, we used the Crime Register that included national complete data on all convictions in lower court from 1973-2018; Swedish Suspicion Register that included national data on individuals strongly suspected of crime from 1998-2018; and the Mortality Register with dates and causes of death from 1952 until 2018.

*ICD codes used to identify alcohol-related medical conditions*

ICD8:

571.0 - Cirrhosis of liver

ICD9:

357F (357.5) Alcoholic polyneuropathy.

425F (425.5) Alcoholic cardiomyopathy

535D (535.3) Alcoholic gastritis

571A (571.0) Alcoholic fatty liver

571B (571.1) Alcoholic hepatitis

571C (571.2) Alcohol cirrhosis liver

571D (571.3) Alcoholic liver damage, unspecified

ICD10:

E24.4: alcohol-induced pseudo-Cushing syndrome

G31.2: alcoholic cardiomyopathy

G62.1: alcoholic neuropathy

G72.1:  myopathy due to alcohol

I42.6: alcoholic cardiomyopathy

K29.2: alcoholic cardiomyopathy

K70.0: fatty liver due to alcohol

K70.1: alcoholic hepatitis

K70.2: liver fibrosis and liver sclerosis due to alcohol

K70.3: liver cirrhosis due to alcohol

K70.4: liver failure due to alcohol

K70.9: unspecified liver injury due to alcohol

K85.2: liver failure due to alcohol

K86.0: chronic pancreatitis due to alcohol

O35.4: Care of pregnant mother with alcohol abuse in which the foetus may be affected

*Statistical analyses*

Analyses were conducted 2022/10/03-2023/09/14. Birth cohort selection and the beginning of follow-up were data-driven and were based on preliminary descriptive analyses to maximize sample size, coverage within the relevant registries, and the proportion of the follow-up period during which individuals were most likely to develop AMC (i.e., beginning follow-up at age 20, when the vast majority of AMC registrations occur much later, would not be ideal). We used Cox proportional hazards models to leverage the longitudinal nature of the data and evaluated whether inclusion of a linear term for time was adequate for subsequent modeling. We elected to use income at age 40, rather than income across time as a time-varying predictor, to facilitate model convergence and to maximize coverage (i.e., follow-up time). We further used preliminary analyses to determine the ages at which “snapshots” of effect sizes would be most relevant to report, and selected (in addition to baseline, time 0) 5 years, 10 years, and 15 years. Although we did not have a formal prospective analysis plan, once we established the above data-driven decisions, all analyses were consistent with our original intentions, with no additional analyses included, to avoid over-interpretation of the data. The models tested are explained sequentially in the primary text.

We used the relative excess risk due to interaction (RERI) and synergy index (S) to estimate additive interactions. The scale for RERI is negative to positive infinity, with 0 indicating no deviation from additivity, a negative estimate indicating less than additivity, and a positive estimate indicating more than additivity. The S can range from 0 to positive infinity, with 1 indicating no deviation from additivity, <1 indicating less than additivity, and >1 indicating more than additivity [1].

1. Knol MJ, VanderWeele TJ, Groenwold RH, Klungel OH, Rovers MM, Grobbee DE. Estimating measures of interaction on an additive scale for preventive exposures. Eur J Epidemiol. 2011;26(6):433-8. Epub 20110223. doi: 10.1007/s10654-011-9554-9. PubMed PMID: 21344323; PubMed Central PMCID: PMC3115067.
