# Supplementary material for: Socioeconomic position indicators and risk of alcohol-related medical conditions: A national cohort study from Sweden
Source: PLoS Med. 2024 Mar 19;21(3):e1004359. doi: 10.1371/journal.pmed.1004359 (PMC10950249; doi:10.1371/journal.pmed.1004359)
Supplement: S1 Table — (DOCX) [file pmed.1004359.s002.docx]

**S1 Table.** Incidence rates of alcohol-related medical conditions, reported as number of new cases per 10,000 person years, for variables that are constant over time.

|  | **Incidences** | |
| --- | --- | --- |
| **Variable** | **Females** | **Males** |
| Crude | 2.01 | 5.20 |
| Marital Status |  |  |
| Married | 1.44 | 3.20 |
| Unmarried | 2.34 | 6.92 |
| Divorced | 3.79 | 9.52 |
| Widowed | 3.21 | 8.03 |
| Educational attainment |  |  |
| Low education | 3.89 | 7.42 |
| Mid education | 2.21 | 5.31 |
| High education | 1.23 | 3.96 |
| Income quartile |  |  |
| Income Quartile 1 | 5.06 | 12.86 |
| Income Quartile 2 | 2.34 | 5.24 |
| Income Quartile 3 | 1.40 | 3.35 |
| Income Quartile 4 | 1.18 | 2.84 |
| Region of origin |  |  |
| Sweden | 2.02 | 5.21 |
| Africa | 0.96 | 3.09 |
| Asia | 0.61 | 2.66 |
| East Europe | 1.68 | 4.32 |
| Finland | 4.22 | 13.24 |
| Latin America | 1.21 | 3.54 |
| Middle East | 0.37 | 2.10 |
| West | 1.96 | 4.18 |
