# Supplementary material for: Socioeconomic position indicators and risk of alcohol-related medical conditions: A national cohort study from Sweden
Source: PLoS Med. 2024 Mar 19;21(3):e1004359. doi: 10.1371/journal.pmed.1004359 (PMC10950249; doi:10.1371/journal.pmed.1004359)
Supplement: S2 Table — Cross-tabulation of income quartile and education level for females in the full sample (top panel) and among those with an alcohol-related medical condition (AMC; bottom panel). (DOCX) [file pmed.1004359.s003.docx]

**S2 Table.** Cross-tabulation of income quartile and education level for females in the full sample (top panel) and among those with an alcohol-related medical condition (AMC; bottom panel).

| Full Female Sample | | | | |
| --- | --- | --- | --- | --- |
|  | Low income | Low-mid income | High-mid income | High income |
| Low education | 24,844 (2.14%) | 36,094 (3.1%) | 38,580 (3.32%) | 26,860 (2.31%) |
| Mid education | 74,473 (6.41%) | 135,227 (11.63%) | 186,585 (16.05%) | 154,946 (13.33%) |
| High education | 51,635 (4.44%) | 103,745 (8.92%) | 126,930 (10.92%) | 202,760 (17.44%) |
|  |  |  |  |  |
| Female sample with AMC | | | | |
|  | Low income | Low-mid income | High-mid income | High income |
| Low education | 371 (1.49%) | 266 (0.74%) | 201 (0.52%) | 145 (0.54%) |
| Mid education | 648 (0.87%) | 625 (0.46%) | 520 (0.28%) | 417 (0.27%) |
| High education | 337 (0.65%) | 278 (0.27%) | 177 (0.14%) | 268 (0.13%) |
