# Supplementary material for: Socioeconomic position indicators and risk of alcohol-related medical conditions: A national cohort study from Sweden
Source: PLoS Med. 2024 Mar 19;21(3):e1004359. doi: 10.1371/journal.pmed.1004359 (PMC10950249; doi:10.1371/journal.pmed.1004359)
Supplement: S3 Table — Cross-tabulation of income quartile and education level for males in the full sample (top panel) and among those with an alcohol-related medical condition (AMC; bottom panel). (DOCX) [file pmed.1004359.s004.docx]

**S3 Table.** Cross-tabulation of income quartile and education level for males in the full sample (top panel) and among those with an alcohol-related medical condition (AMC; bottom panel).

| Full Male Sample | | | | |
| --- | --- | --- | --- | --- |
|  | Low income | Low-mid income | High-mid income | High income |
| Low education | 42,091 (3.52%) | 50,847 (4.25%) | 62,532 (5.23%) | 33,262 (2.78%) |
| Mid education | 96,157 (8.04%) | 148,555 (12.41%) | 202,873 (16.95%) | 132,796 (11.1%) |
| High education | 61,157 (5.11%) | 84,951 (7.1%) | 110,009 (9.19%) | 171,429 (14.33%) |
|  |  |  |  |  |
| Male sample with AMC | | | | |
|  | Low income | Low-mid income | High-mid income | High income |
| Low education | 42,091 (3.52%) | 50,847 (4.25%) | 62,532 (5.23%) | 33,262 (2.78%) |
| Mid education | 96,157 (8.04%) | 148,555 (12.41%) | 202,873 (16.95%) | 132,796 (11.1%) |
| High education | 61,157 (5.11%) | 84,951 (7.1%) | 110,009 (9.19%) | 171,429 (14.33%) |
