# Supplementary material for: Socioeconomic position indicators and risk of alcohol-related medical conditions: A national cohort study from Sweden
Source: PLoS Med. 2024 Mar 19;21(3):e1004359. doi: 10.1371/journal.pmed.1004359 (PMC10950249; doi:10.1371/journal.pmed.1004359)
Supplement: S4 Table — Hazard ratios, 95% confidence intervals, and p-values from Chi-square tests are presented. The primary predictor of interest (here, education level) was modeled using a time-varying coefficient, with a linear term for time. Below, we provide snapshots of hazard ratios for education at 4 time points: at the beginning of observation (time 0), after 5 years, after 10 years, and after 15 years. (DOCX) [file pmed.1004359.s005.docx]

**S4 Table.** Complete results for Model 1A for females and males, testing the association between education level and alcohol-related medical conditions. Hazard ratios, 95% confidence intervals, and p-values from Chi-square tests are presented. The primary predictor of interest (here, education level) was modeled using a time-varying coefficient, with a linear term for time. Below, we provide snapshots of hazard ratios for education at four timepoints: at the beginning of observation (time 0), after 5 years, after 10 years, and after 15 years.

|  | *Females* | | | | *Males* | | | |
| --- | --- | --- | --- | --- | --- | --- | --- | --- |
| *Variable* | Time 0 | 5 years | 10 years | 15 years | Time 0 | 5 years | 10 years | 15 years |
| Education low vs. high | 4.67  (3.82, 5.72); p<0.001 | 3.96  (3.42, 4.58); p<0.001 | 3.35  (3.03, 3.71); p<0.001 | 2.84  (2.60, 3.11); p<0.001 | 2.04  (1.81, 2.30); p<0.001 | 1.87  (1.72, 2.04); p<0.001 | 1.72  (1.62, 1.82); p<0.001 | 1.57  (1.49, 1.66); p<0.001 |
| Education mid vs. high | 1.88  (1.58, 2.23), p<0.001 | 1.81  (1.60, 2.04); p<0.001 | 1.74  (1.60, 1.90); p<0.001 | 1.68  (1.56, 1.81); p<0.001 | 1.33  (1.20, 1.48); p<0.001 | 1.31  (1.22, 1.41); p<0.001 | 1.28  (1.22, 1.35); p<0.001 | 1.26  (1.20, 1.32); p<0.001 |
| Birth year | 1.01 (1.01, 1.02); p<0.001 | | | | 0.997 (0.992, 1.001; p=0.100 | | | |
| Marital status |  | | | |  | | | |
| Married | Reference | | | | Reference | | | |
| Unmarried | 1.61 (1.98, 1.72); p<0.001 | | | | 2.22 (2.13, 2.31); p<0.001 | | | |
| Divorced | 2.57 (2.38, 2.78); p<0.001 | | | | 3.02 (1.86, 3.19); p<0.001 | | | |
| Widowed | 2.05 (1.50, 2.79); p<0.001 | | | | 2.38 (1.65, 3.43); p<0.001 | | | |
| Region of origin |  | | | |  | | | |
| Sweden | Reference | | | | Reference | | | |
| Africa | 0.38 (0.20, 0.73); p=0.004 | | | | 0.66 (0.48, 0.91); p=0.656 | | | |
| Asia | 0.25 (0.16, 0.38); p<0.001 | | | | 0.60 (0.47, 0.76); p<0.001 | | | |
| East Europe | 0.76 (0.64, 0.91); p=0.002 | | | | 0.96 (0.84, 1.09); p=0.562 | | | |
| Finland | 1.74 (1.54, 1.97); p<0.001 | | | | 2.16 (2.02, 2.37); p<0.001 | | | |
| Latin America | 0.50 (0.32, 0.77); p=0.002 | | | | 0.64 (0.49, 0.83); p=0.001 | | | |
| Middle East | 0.18 (0.11, 0.29); p<0.001 | | | | 0.48 (0.40, 0.57); p<0.001 | | | |
| Western Europe | 0.94 (0.75, 1.18); p=0.594 | | | | 0.83 (0.72, 0.97); p=0.016 | | | |
