# Supplementary material for: Socioeconomic position indicators and risk of alcohol-related medical conditions: A national cohort study from Sweden
Source: PLoS Med. 2024 Mar 19;21(3):e1004359. doi: 10.1371/journal.pmed.1004359 (PMC10950249; doi:10.1371/journal.pmed.1004359)
Supplement: S5 Table — Hazard ratios, 95% confidence intervals, and p-values from Chi-square tests are presented. The primary predictor of interest (here, income) was modeled using a time-varying coefficient, with a linear term for time. Below, we provide snapshots of hazard ratios for income at 4 time points: at the beginning of observation (time 0), after 5 years, after 10 years, and after 15 years. (DOCX) [file pmed.1004359.s006.docx]

**S5 Table.** Complete results for Model 1B for females and males, testing the association between income and alcohol-related medical conditions. Hazard ratios, 95% confidence intervals, and p-values from Chi-square tests are presented. The primary predictor of interest (here, income) was modeled using a time-varying coefficient, with a linear term for time. Below, we provide snapshots of hazard ratios for income at four timepoints: at the beginning of observation (time 0), after 5 years, after 10 years, and after 15 years.

|  | *Females* | | | | *Males* | | | |
| --- | --- | --- | --- | --- | --- | --- | --- | --- |
| *Variable* | Time 0 | 5 years | 10 years | 15 years | Time 0 | 5 years | 10 years | 15 years |
| Income quartile  1 vs. 4 | 11.26  (9.03, 14.05); p<0.001 | 7.88  (6.69, 9.29); p<0.001 | 5.52  (4.89, 6.22); p<0.001 | 3.86  (3.47, 4.30); p<0.001 | 10.21  (8.86, 11.77); p<0.001 | 7.14  (6.43, 7.49); p<0.001 | 5.00  (4.63, 5.40); p<0.001 | 3.50  (3.27, 3.74); p<0.001 |
| Income quartile  2 vs. 4 | 3.70  (2.95, 4.65); p<0.001 | 2.92  (2.47, 3.46); p<0.001 | 2.31  (2.04, 2.60); p<0.001 | 1.82  (1.64, 2.01); p<0.001 | 2.90  (2.49, 3.38); p<0.001 | 2.38  (2.12, 2.66); p<0.001 | 1.95  (1.80, 2.11); p<0.001 | 1.59  (1.49, 1.70); p<0.001 |
| Income quartile  3 vs. 4 | 1.62  (1.28, 2.06); p<0.001 | 1.47  (1.24, 1.75); p<0.001 | 1.33  (1.18, 1.51); p<0.001 | 1.21  (1.10, 1.33); p<0.001 | 1.59  (1.36, 1.86); p<0.001 | 1.43  (1.28, 1.61); p<0.001 | 1.29  (1.19, 1.39); p<0.001 | 1.16  (1.09, 1.23) ; p<0.001 |
| Birth year | 1.01 (1.00, 1.02); p<0.001 | | | | 1.00 (1.00, 1.00); p=0.770 | | | |
| Marital status |  | | | |  | | | |
| Married | Reference | | | | Reference | | | |
| Unmarried | 0.92 (0.85, 1.01); p=0.073 | | | | 1.32 (1.26, 1.39); p<0.001 | | | |
| Divorced | 1.52 (1.39, 1.67); p<0.001 | | | | 1.71 (1.61, 1.82); p<0.001 | | | |
| Widowed | 1.55 (1.14, 2.12); p=0.006 | | | | 1.83 (1.27, 2.64); p=0.001 | | | |
| Region of origin |  | | | |  | | | |
| Sweden | Reference | | | | Reference | | | |
| Africa | 0.39 (0.20, 0.74); p=0.004 | | | | 0.43 (0.31, 0.59); | | | |
| Asia | 0.25 (0.17, 0.39); p<0.001 | | | | 0.44 (0.35, 0.56); p<0.001 | | | |
| East Europe | 0.66 (0.55, 0.78); p<0.001 | | | | 0.73 (0.64, 0.83); p<0.001 | | | |
| Finland | 1.80 (1.59, 2.03); p<0.001 | | | | 2.06 (1.90, 2.23); p<0.001 | | | |
| Latin America | 0.48 (0.31, 0.75); p=0.001 | | | | 0.48 (0.37, 0.63); p<0.001 | | | |
| Middle East | 0.15 (0.09, 0.25); p<0.001 | | | | 0.31 (0.26, 0.37); p<0.001 | | | |
| Western Europe | 0.87 (0.69, 1.09); p=0.236 | | | | 0.71 (0.61, 0.83); p<0.001 | | | |
