# Supplementary material for: Socioeconomic position indicators and risk of alcohol-related medical conditions: A national cohort study from Sweden
Source: PLoS Med. 2024 Mar 19;21(3):e1004359. doi: 10.1371/journal.pmed.1004359 (PMC10950249; doi:10.1371/journal.pmed.1004359)
Supplement: S6 Table — Hazard ratios, 95% confidence intervals, and p-values from Chi-square tests are presented. The primary predictors of interest (education level and income) were modeled using time-varying coefficients, with a linear term for time. Below, we provide snapshots of hazard ratios for education and income at 4 time points: at the beginning of observation (time 0), after 5 years, after 10 years, and after 15 years. (DOCX) [file pmed.1004359.s007.docx]

**S6 Table.** Complete results for Model 2 for females and males, testing the associations between education level and income with alcohol-related medical conditions. Hazard ratios, 95% confidence intervals, and p-values from Chi-square tests are presented. The primary predictors of interest (education level and income) were modeled using time-varying coefficients, with a linear term for time. Below, we provide snapshots of hazard ratios for education and income at four timepoints: at the beginning of observation (time 0), after 5 years, after 10 years, and after 15 years.

|  | *Females* | | | | *Males* | | | |
| --- | --- | --- | --- | --- | --- | --- | --- | --- |
| *Variable* | Time 0 | 5 years | 10 years | 15 years | Time 0 | 5 years | 10 years | 15 years |
| Education  low vs. high | 3.51  (2.86, 4.30); p<0.001 | 3.15  (2.73, 3.54); p<0.001 | 2.84  (2.56, 3.14); p<0.001 | 2.55  (2.33, 2.80); p<0.001 | 1.63  (1.43, 1.83); p<0.001 | 1.56  (1.43, 1.70); p<0.001 | 1.50  (1.41, 1.59); p<0.001 | 1.45  (1.37, 1.53); p<0.001 |
| Education  mid vs. high | 1.67  (1.41, 1.99); p<0.001 | 1.65  (1.46, 1.87); p<0.001 | 1.63  (1.49, 1.77); p<0.001 | 1.60  (1.49, 1.73); p<0.001 | 1.20  (1.08, 1.33); p<0.001 | 1.20  (1.09, 1.32); p<0.001 | 1.20  (1.12, 1.29); p<0.001 | 1.20  (1.14, 1.27); p<0.001 |
| Income quartile  1 vs. 4 | 9.24  (7.39, 11.54); p<0.001 | 6.60  (5.59, 7.78); p<0.001 | 4.71  (4.17, 5.32); p<0.001 | 3.36  (3.02, 3.75); p<0.001 | 9.74  (8.21, 10.93); p<0.001 | 6.66  (5.99, 7.40); p<0.001 | 4.68  (4.34, 5.06); p<0.001 | 3.29  (3.08, 3.52); p<0.001 |
| Income quartile  2 vs. 4 | 3.25  (2.59, 4.08); p<0.001 | 2.59  (2.19, 3.07); p<0.001 | 2.07  (1.84, 2.33); p<0.001 | 1.65  (1.49, 1.83); p<0.001 | 2.72  (2.34, 3.17); p<0.001 | 2.24  (2.00, 2.50); p<0.001 | 1.84  (1.70, 1.99); p<0.001 | 1.51  (1.41, 1.61); p<0.001 |
| Income quartile  3 vs. 4 | 1.45  (1.14, 1.84); p=0.014 | 1.32  (1.11, 1.57); p=0.002 | 1.20  (1.07, 1.36); p=0.003 | 1.10  (1.00, 1.21); p=0.057 | 1.50  (1.28, 1.76); p<0.001 | 1.35  (1.21, 1.52); p<0.001 | 1.22  (1.13, 1.32); p<0.001 | 1.10  (1.03, 1.17); p=0.004 |
| Birth year | 1.02 (1.01, 1.02); p<0.001 | | | | 1.00 (1.00, 1.01); p=0.155 | | | |
| Marital status |  | | | |  | | | |
| Married | Reference | | | | Reference | | | |
| Unmarried | 0.93 (0.86, 1.02); p=0.130 | | | | 1.33 (1.26, 1.39); p<0.001 | | | |
| Divorced | 1.52 (1.39, 1.67); p<0.001 | | | | 1.71 (1.61, 1.82); p<0.001 | | | |
| Widowed | 1.48 (1.09, 2.03); p=0.013 | | | | 1.81 (1.26, 2.62); p<0.001 | | | |
| Regio of origin |  | | | |  | | | |
| Sweden | Reference | | | | Reference | | | |
| Africa | 0.34 (0.18, 0.65); p=0.001 | | | | 0.44 (0.32, 0.61); p<0.001 | | | |
| Asia | 0.21 (0.14, 0.32); p<0.001 | | | | 0.43 (0.34, 0.55); p<0.001 | | | |
| East Europe | 0.65 (0.54, 0.77); p<0.001 | | | | 0.75 (0.66, 0.85); p<0.001 | | | |
| Finland | 1.72 (1.52, 1.95); p<0.001 | | | | 2.02 (1.87, 2.19); p<0.001 | | | |
| Latin America | 0.47 (0.30, 0.73); p<0.001 | | | | 0.49 (0.38, 0.64); p<0.001 | | | |
| Middle East | 0.14 (0.09, 0.24); p<0.001 | | | | 0.32 (0.27, 0.38); p<0.001 | | | |
| Western Europe | 0.87 (0.70, 1,10); p=0.242 | | | | 0.73 (0.63, 0.85); p<0.001 | | | |
