# Supplementary material for: Socioeconomic position indicators and risk of alcohol-related medical conditions: A national cohort study from Sweden
Source: PLoS Med. 2024 Mar 19;21(3):e1004359. doi: 10.1371/journal.pmed.1004359 (PMC10950249; doi:10.1371/journal.pmed.1004359)
Supplement: S7 Table — Hazard ratios, 95% confidence intervals, and Chi-square p-values are presented. The primary predictors of interest (education level and income) were modeled using time-varying coefficients, with a linear term for time. Below, we provide snapshots of hazard ratios for education and income at 4 time points: at the beginning of observation (time 0), after 5 years, after 10 years, and after 15 years. (DOCX) [file pmed.1004359.s008.docx]

**S7 Table.** Complete results for Model 3 for females and males, testing the associations between education level and income with alcohol-related medical conditions. Hazard ratios, 95% confidence intervals, and Chi-square p-values are presented. The primary predictors of interest (education level and income) were modeled using time-varying coefficients, with a linear term for time. Below, we provide snapshots of hazard ratios for education and income at four timepoints: at the beginning of observation (time 0), after 5 years, after 10 years, and after 15 years.

|  | *Females* | | | | *Males* | | | |
| --- | --- | --- | --- | --- | --- | --- | --- | --- |
| *Variable* | Time 0 | 5 years | 10 years | 15 years | Time 0 | 5 years | 10 years | 15 years |
| Education  low vs. high | 3.17  (2.58, 3.88); p<0.001 | 2.87  (2.48, 3.32); p<0.001 | 2.60  (2.38, 2.86); p<0.001 | 2.36  (2.15, 2.58); p<0.001 | 1.63  (1.44, 1.83); p<0.001 | 1.57  (1.44, 1.71); p<0.001 | 1.51  (1.43, 1.61); p<0.001 | 1.46  (1.39, 1.54); p<0.001 |
| Education  mid vs. high | 1.61  (1.35, 1.91); p<0.001 | 1.59  (1.40, 1.80); p<0.001 | 1.57  (1.44, 1.71); p<0.001 | 1.55  (1.44, 1.67); p<0.001 | 1.21  (1.09, 1.34); p<0.001 | 1.21  (1.13, 1.30); p<0.001 | 1.21  (1.15, 1.28); p<0.001 | 1.21  (1.16, 1.27); p<0.001 |
| Income quartile  1 vs. 4 | 7.80  (6.24, 9.75); p<0.001 | 5.59  (4.74, 6.60); p<0.001 | 4.01  (3.56, 4.53); p<0.001 | 2.88  (2.58, 3.20); p<0.001 | 7.76  (6.73, 8.95); p<0.001 | 5.48  (4.93, 6.09); p<0.001 | 3.87  (3.58, 4.18); p<0.001 | 2.73  (2.55, 2.92); p<0.001 |
| Income quartile  2 vs. 4 | 2.92  (2.33, 3.67); p<0.001 | 2.34  (1.98, 2.77); p<0.001 | 1.88  (1.66, 2.11); p<0.001 | 1.50  (1.36, 1.66); p<0.001 | 2.50  (2.15, 2.91); p<0.001 | 2.06  (1.84, 2.31); p<0.001 | 1.70  (1.57, 1.84); p<0.001 | 1.40  (1.31, 1.46); p<0.001 |
| Income quartile  3 vs. 4 | 1.39  (1.09, 1.76); p=0.007 | 1.27  (1.07, 1.52); p=0.007 | 1.17  (1.03, 1.31); p=0.013 | 1.07  (0.97, 1.17); p=0.181 | 1.44  (1.23, 1.69); p<0.001 | 1.30  (1.16, 1.46); p<0.001 | 1.18  (1.09, 1.27); p<0.001 | 1.06  (1.00, 1.13); p=0.065 |
| Birth year | 1.02 (1.02, 1.03); p<0.001 | | | | 1.01 (1.00, 1.01); p<0.001 | | | |
| Marital status |  | | | |  | | | |
| Married | Reference | | | | Reference | | | |
| Unmarried | 0.92 (0.84, 1.00); p=0.059 | | | | 1.32 (1.25, 1.39); p<0.001 | | | |
| Divorced | 1.35 (1.23, 1.48); p<0.001 | | | | 1.63 (1.53, 1.74); p<0.001 | | | |
| Widowed | 1.34 (0.98, 1.84); p=0.063 | | | | 1.67 (1.16, 2.41); p=0.006 | | | |
| Region of origin |  | | | |  | | | |
| Sweden | Reference | | | | Reference | | | |
| Africa | 0.37 (0.19, 0.71); p=0.003 | | | | 0.45 (0.32, 0.62); p<0.001 | | | |
| Asia | 0.21 (0.14, 0.32); p<0.001 | | | | 0.40 (0.31, 0.50); p<0.001 | | | |
| East Europe | 0.53 (0.44, 0.63); p<0.001 | | | | 0.64 (0.56, 0.72); p<0.001 | | | |
| Finland | 1.70 (1.50, 1.92); p<0.001 | | | | 2.03 (1.87, 2.20); p<0.001 | | | |
| Latin America | 0.38 (0.24, 0.59); p<0.001 | | | | 0.45 (0.34, 0.59); p<0.001 | | | |
| Middle East | 0.11 (0.07, 0.18); p<0.001 | | | | 0.26 (0.22, 0.31); p<0.001 | | | |
| Western Europe | 0.83 (0.66, 1.04); p=0.106 | | | | 0.69 (0.60, 0.81); p<0.001 | | | |
| Internalizing disorders | 2.64 (2.48, 2.81); p<0.001 | | | | 3.18 (3.06, 3.31); p<0.001 | | | |
| Externalizing disorders | 3.71 (3.40, 4.04); p<0.003 | | | | 2.15 (2.02, 2.28); p<0.001 | | | |
