# Supplementary material for: Socioeconomic position indicators and risk of alcohol-related medical conditions: A national cohort study from Sweden
Source: PLoS Med. 2024 Mar 19;21(3):e1004359. doi: 10.1371/journal.pmed.1004359 (PMC10950249; doi:10.1371/journal.pmed.1004359)
Supplement: S8 Table — Hazard ratios, 95% confidence intervals, and Chi-square p-values are presented. The primary predictors of interest (education level and income) were modeled using time-varying coefficients, with a linear term for time. Below, we provide snapshots of hazard ratios for education and income at 4 time points: at the beginning of observation (time 0), after 5 years, after 10 years, and after 15 years. (DOCX) [file pmed.1004359.s009.docx]

**S8 Table.** Complete results for Model 4 for females and males, testing the associations between education level and income with alcohol-related medical conditions. Hazard ratios, 95% confidence intervals, and Chi-square p-values are presented. The primary predictors of interest (education level and income) were modeled using time-varying coefficients, with a linear term for time. Below, we provide snapshots of hazard ratios for education and income at four timepoints: at the beginning of observation (time 0), after 5 years, after 10 years, and after 15 years.

|  | *Females* | | | | *Males* | | | |
| --- | --- | --- | --- | --- | --- | --- | --- | --- |
| *Variable* | Time 0 | 5 years | 10 years | 15 years | Time 0 | 5 years | 10 years | 15 years |
| Education  low vs. high | 2.46  (2.00, 3.01); p<0.001 | 2.28  (1.97, 2.64); p<0.001 | 2.12  (1.92, 2.35); p<0.001 | 1.97  (1.80, 2.16); p<0.001 | 1.48  (1.31, 1.67); p<0.001 | 1.43  (1.31, 1.56); p<0.001 | 1.39  (1.31, 1.47); p<0.001 | 1.34  (1.27, 1.42); p<0.001 |
| Education  mid vs. high | 1.42  (1.19, 1.69); p<0.001 | 1.41  (1.25, 1.60); p<0.001 | 1.41  (1.30, 1.54); p<0.001 | 1.40  (1.31, 1.52); p<0.001 | 1.14  (1.03, 1.27); p=0.011 | 1.14  (1.06, 1.23); p<0.001 | 1.14  (1.08, 1.20); p<0.001 | 1.13  (1.08, 1.19); p<0.001 |
| Income quartile  1 vs. 4 | 5.86  (4.69, 7.32); p<0.001 | 4.33  (3.67, 5.11); p<0.001 | 3.21  (2.84, 3.62); p<0.001 | 2.37  (2.13, 2.64); p<0.001 | 6.41  (5.56, 7.39); p<0.001 | 4.57  (4.11, 5.08); p<0.001 | 3.26  (3.01, 3.52); p<0.001 | 2.32  (2.17, 2.48); p<0.001 |
| Income quartile  2 vs. 4 | 2.52  (2.01, 3.17); p<0.001 | 2.06  (1.75, 2.44); p<0.001 | 1.69  (1.50, 1.90); p<0.001 | 1.38  (1.25, 1.53); p<0.001 | 2.30  (1.97, 2.68); p<0.001 | 1.91  (1.71, 2.14); p<0.001 | 1.59  (1.47, 1.72); p<0.001 | 1.33  (1.24, 1.42); p<0.001 |
| Income quartile  3 vs. 4 | 1.38  (1.09, 1.76); p=0.008 | 1.28  (1.08, 1.53); p=0.006 | 1.19  (1.05, 1.34); p=0.006 | 1.10  (1.00, 1.21); p=0.057 | 1.41  (1.21, 1.65); p<0.001 | 1.28  (1.14, 1.44); p<0.001 | 1.17  (1.08, 1.27); p<0.001 | 1.07  (1.00, 1.13); p=0.051 |
| Birth year | 1.03 (1.02, 1.04); p<0.001 | | | | 1.02 (1.02, 1.03); p<0.001 | | | |
| Marital status |  | | | |  | | | |
| Married | Reference | | | | Reference | | | |
| Unmarried | 0.87 (0.80, 0.95); p=0.002 | | | | 1.25 (1.19, 1.31); p<0.001 | | | |
| Divorced | 1.08 (0.99, 1.19); p=0.092 | | | | 1.41 (1.32, 1.50); p<0.001 | | | |
| Widowed | 1.10 (0.80, 1.50); p=0.561 | | | | 1.59 (1.11, 2.30); p=0.013 | | | |
| Region of origin |  | | | |  | | | |
| Sweden | Reference | | | | Reference | | | |
| Africa | 0.60 (0.31, 1.15); p=0.121 | | | | 0.55 (0.40, 0.76); p<0.001 | | | |
| Asia | 0.37 (0.24, 0.57); p<0.001 | | | | 0.52 (0.41, 0.65); p<0.001 | | | |
| East Europe | 0.81 (0.68, 0.96); p=0.016 | | | | 0.75 (0.66, 0.85); p<0.001 | | | |
| Finland | 1.26 (1.11, 1.42); p<0.001 | | | | 1.67 (1.54, 1.81); p<0.001 | | | |
| Latin America | 0.61 (0.40, 0.95); p=0.030 | | | | 0.54 (0.41, 0.71); p<0.001 | | | |
| Middle East | 0.27 (0.16, 0.46); p<0.001 | | | | 0.38 (0.32, 0.46); p<0.001 | | | |
| Western Europe | 0.89 (0.71, 1.11); p=0.295 | | | | 0.76 (0.65, 0.88); p<0.001 | | | |
| Internalizing disorders | 1.28 (1.20, 1.37); p<0.001 | | | | 1.85 (1.78, 1.93); p<0.001 | | | |
| Externalizing disorders | 1.27 (1.16, 1.38); p<0.001 | | | | 1.25 (1.18, 1.33); p<0.001 | | | |
| Alcohol use disorder | 34.00 (31.73, 36.42); p<0.001 | | | | 10.71 (10.28, 11.15); p<0.001 | | | |
