# Supplementary material for: Socioeconomic position indicators and risk of alcohol-related medical conditions: A national cohort study from Sweden
Source: PLoS Med. 2024 Mar 19;21(3):e1004359. doi: 10.1371/journal.pmed.1004359 (PMC10950249; doi:10.1371/journal.pmed.1004359)
Supplement: S9 Table — Hazard ratios, 95% confidence intervals, and Chi-square p-values are presented. The primary predictor of interest (here, education level) was modeled using a time-varying coefficient, with a linear term for time. Below, we provide snapshots of hazard ratios for education at 4 time points: at the beginning of observation (time 0), after 5 years, after 10 years, and after 15 years. These secondary analyses were limited to the subsample born in Sweden with 2 Swedish-born parents to improve the precision of the family genetic risk score for alcohol use disorder; accordingly, region of interest is excluded as a covariate. (DOCX) [file pmed.1004359.s010.docx]

**S9 Table.** Complete results for Model S1A for females and males, testing the association between education level and alcohol-related medical conditions. Hazard ratios, 95% confidence intervals, and Chi-square p-values are presented. The primary predictor of interest (here, education level) was modeled using a time-varying coefficient, with a linear term for time. Below, we provide snapshots of hazard ratios for education at four timepoints: at the beginning of observation (time 0), after 5 years, after 10 years, and after 15 years. These secondary analyses were limited to the subsample born in Sweden with two Swedish-born parents to improve the precision of the family genetic risk score for alcohol use disorder; accordingly, region of interest is excluded as a covariate.

|  | *Females* | | | | *Males* | | | |
| --- | --- | --- | --- | --- | --- | --- | --- | --- |
| *Variable* | Time 0 | 5 years | 10 years | 15 years | Time 0 | 5 years | 10 years | 15 years |
| Education  low vs. high | 3.78  (2.98, 4.81); p<0.001 | 3.36  (2.82, 3.99); p<0.001 | 2.98  (2.64, 3.35); p<0.001 | 2.64  (2.38, 2.92); p<0.001 | 1.95  (1.70, 2.25); p<0.001 | 1.80  (1.63, 1.99); p<0.001 | 1.66  (1.54, 1.77); p<0.001 | 1.52  (1.43, 1.62); p<0.001 |
| Education  mid vs. high | 1.77  (1.45, 2.16); p<0.001 | 1.72  (1.49, 1.98); p<0.001 | 1.66  (1.51, 1.84); p<0.001 | 1.61  (1.48, 1.76); p<0.001 | 1.33  (1.18, 1.50); p<0.001 | 1.30  (1.19, 1.42); p<0.001 | 1.27  (1.20, 1.35); p<0.001 | 1.24  (1.18, 1.31); p<0.001 |
| Birth year | 1.01 (1.00, 1.02); p=0.001 | | | | 1.00 (0.99, 1.00); p=0.129 | | | |
| Marital status |  | | | |  | | | |
| Married | Reference | | | | Reference | | | |
| Unmarried | 1.54 (1.42, 1.67); p<0.001 | | | | 2.07 (1.97, 2.17); p<0.001 | | | |
| Divorced | 2.43 (1.22, 2.66); p<0.001 | | | | 2.81 (2.63, 2.99); p<0.001 | | | |
| Widowed | 2.01 (1.40, 2.89); p<0.001 | | | | 2.69 (1.84, 3.93); p<0.001 | | | |
| FGRS_AUD_ | 1.38 (1.35, 1.41); p<0.001 | | | | 1.36 (1.34, 1.38); p<0.001 | | | |

FGRS_AUD_ = family genetic risk score for alcohol use disorder
