# Supplementary material for: Socioeconomic position indicators and risk of alcohol-related medical conditions: A national cohort study from Sweden
Source: PLoS Med. 2024 Mar 19;21(3):e1004359. doi: 10.1371/journal.pmed.1004359 (PMC10950249; doi:10.1371/journal.pmed.1004359)
Supplement: S10 Table — Hazard ratios and 95% confidence intervals are presented. The primary predictor of interest (here, income) was modeled using a time-varying coefficient, with a linear term for time. Below, we provide snapshots of hazard ratios for income at 4 time points: at the beginning of observation (time 0), after 5 years, after 10 years, and after 15 years. These secondary analyses were limited to the subsample born in Sweden with 2 Swedish-born parents to improve the precision of the family genetic risk score for alcohol use disorder; accordingly, region of interest is excluded as a covariate. (DOCX) [file pmed.1004359.s011.docx]

**S10 Table.** Complete results for Model S1B for females and males, testing the association between income and alcohol-related medical conditions. Hazard ratios and 95% confidence intervals are presented. The primary predictor of interest (here, income) was modeled using a time-varying coefficient, with a linear term for time. Below, we provide snapshots of hazard ratios for income at four timepoints: at the beginning of observation (time 0), after 5 years, after 10 years, and after 15 years. These secondary analyses were limited to the subsample born in Sweden with two Swedish-born parents to improve the precision of the family genetic risk score for alcohol use disorder; accordingly, region of interest is excluded as a covariate.

|  | *Females* | | | | *Males* | | | |
| --- | --- | --- | --- | --- | --- | --- | --- | --- |
| *Variable* | Time 0 | 5 years | 10 years | 15 years | Time 0 | 5 years | 10 years | 15 years |
| Income quartile  1 vs. 4 | 9.57  (7.44,12.32); p<0.001 | 6.97  (5.78, 8.41); p<0.001 | 5.08  (4.42, 5.83); p<0.001 | 3.70  (3.27, 4.18); p<0.001 | 8.57  (7.31,10.04); p<0.001 | 6.13  (5.45, 6.89); p<0.001 | 4.38  (4.02, 4.78); p<0.001 | 3.14  (2.91, 3.38); p<0.001 |
| Income quartile  2 vs. 4 | 3.38  (2.61, 4.37); p<0.001 | 2.71  (2.24, 3.29); p<0.001 | 2.18  (1.90, 2.50); p<0.001 | 1.75  (1.56, 1.97); p<0.001 | 2.70  (2.28, 3.19); p<0.001 | 2.21  (1.95, 2.51); p<0.001 | 1.82  (1.66, 1.99); p<0.001 | 1.49  (1.39, 1.61); p<0.001 |
| Income quartile  3 vs. 4 | 1.47  (1.12, 1.93); p=0.006 | 1.36  (1.11, 1.66); p=0.003 | 1.26  (1.10, 1.44); p=0.001 | 1.16  (1.05, 1.30); p=0.005 | 1.48  (1.25, 1.77); p<0.001 | 1.35  (1.18, 1.53); p<0.001 | 1.22  (1.12, 1.33); p<0.001 | 1.11  (1.03, 1.19); p=0.004 |
| Birth year | 1.01 (1.00, 1.02); p=0.013 | | | | 1.00 (0.99, 1.00); p=0.982 | | | |
| Marital status |  | | | |  | | | |
| Married | Reference | | | | Reference | | | |
| Unmarried | 0.91 (0.82, 1.00); p=0.053 | | | | 1.30 (1.23, 1.38); p<0.001 | | | |
| Divorced | 1.46 (1.31, 1.62); p<0.001 | | | | 1.68 (1.56, 1.80); p<0.001 | | | |
| Widowed | 1.54 (1.07, 2.22); p=0.019 | | | | 2.22 (1.52, 3.25); p<0.001 | | | |
| FGRS_AUD_ | 1.39 (1.36, 1.42); p<0.001 | | | | 1.33 (1.32, 1.35); p<0.001 | | | |

FGRS_AUD_ = family genetic risk score for alcohol use disorder
