# Supplementary material for: Socioeconomic position indicators and risk of alcohol-related medical conditions: A national cohort study from Sweden
Source: PLoS Med. 2024 Mar 19;21(3):e1004359. doi: 10.1371/journal.pmed.1004359 (PMC10950249; doi:10.1371/journal.pmed.1004359)
Supplement: S11 Table — Hazard ratios, 95% confidence intervals, and Chi-square p-values are presented. The primary predictors of interest (education level and income) were modeled using time-varying coefficients, with a linear term for time. Below, we provide snapshots of hazard ratios for education level and income at 4 time points: at the beginning of observation (time 0), after 5 years, after 10 years, and after 15 years. These secondary analyses were limited to the subsample born in Sweden with 2 Swedish-born parents to improve the precision of the family genetic risk score for alcohol use disorder; accordingly, region of interest is excluded as a covariate. (DOCX) [file pmed.1004359.s012.docx]

**S11 Table.** Complete results for Model S2 for females and males, testing the associations between education level and income with alcohol-related medical conditions. Hazard ratios, 95% confidence intervals, and Chi-square p-values are presented. The primary predictors of interest (education level and income) were modeled using time-varying coefficients, with a linear term for time. Below, we provide snapshots of hazard ratios for education level and income at four timepoints: at the beginning of observation (time 0), after 5 years, after 10 years, and after 15 years. These secondary analyses were limited to the subsample born in Sweden with two Swedish-born parents to improve the precision of the family genetic risk score for alcohol use disorder; accordingly, region of interest is excluded as a covariate.

|  | *Females* | | | | *Males* | | | |
| --- | --- | --- | --- | --- | --- | --- | --- | --- |
| *Variable* | Time 0 | 5 years | 10 years | 15 years | Time 0 | 5 years | 10 years | 15 years |
| Education  low vs. high | 2.83  (2.22, 3.60); p<0.001 | 2.66  (2.24, 3.17); p<0.001 | 2.51  (2.22, 2.83); p<0.001 | 2.36  (2.13, 2.62); p<0.001 | 1.56  (1.36, 1.80); p<0.001 | 1.51  (1.37, 1.67); p<0.001 | 1.46  (1.36, 1.57); p<0.001 | 1.42  (1.33, 1.51); p<0.001 |
| Education  mid vs. high | 1.59  (1.30, 1.94); p<0.001 | 1.57  (1.36, 1.82); p<0.001 | 1.56  (1.41, 1.72); p<0.001 | 1.54  (1.42, 1.68); p<0.001 | 1.20  (1.07, 1.36); p=0.003 | 1.20  (1.10, 1.31); p<0.001 | 1.20  (1.13, 1.28); p<0.001 | 1.21  (1.14, 1.27); p<0.001 |
| Income quartile  1 vs. 4 | 8.09  (6.27,10.44); p<0.001 | 5.97  (4.94, 7.21); p<0.001 | 4.40  (3.83, 5.05); p<0.001 | 3.24  (2.87, 3.66); p<0.001 | 7.98  (6.80, 9.36); p<0.001 | 5.73  (5.09, 6.46); p<0.001 | 4.12  (3.78, 4.49); p<0.001 | 2.96  (2.75, 3.19); p<0.001 |
| Income quartile  2 vs. 4 | 3.03  (2.34, 3.93); p<0.001 | 2.45  (2.02, 2.97); p<0.001 | 1.98  (1.73, 2.27); p<0.001 | 1.60  (1.43, 1.80); p<0.001 | 2.54  (2.14, 3.01); p<0.001 | 2.09  (1.84, 2.37); p<0.001 | 1.72  (1.57, 1.88); p<0.001 | 1.42  (1.33, 1.53); p<0.001 |
| Income quartile  3 vs. 4 | 1.34  (1.02, 1.76); p=0.037 | 1.24  (1.02, 1.51); p=0.035 | 1.15  (1.00, 1.32); p=0.046 | 1.07  (0.96, 1.19); p=0.245 | 1.40  (1.18, 1.67); p<0.001 | 1.27  (1.12, 1.45); p<0.001 | 1.16  (1.06, 1.26); p=0.001 | 1.05  (0.98, 1.13); p=0.163 |
| Birth year | 1.02 (1.01, 1.02); p<0.001 | | | | 1.00 (1.00, 1.01); p=0.449 | | | |
| Marital status |  | | | |  | | | |
| Married | Reference | | | | Reference | | | |
| Unmarried | 0.92 (0.83, 1.01); p=0.084 | | | | 1.30 (1.21, 1.38); p<0.001 | | | |
| Divorced | 1.46 (1.31, 1.62); p<0.001 | | | | 1.68 (1.55, 1.81); p<0.001 | | | |
| Widowed | 1.48 (1.03, 2.13); p=0.034 | | | | 2.20 (1.49, 3.22); p<0.001 | | | |
| FGRS_AUD_ | 1.37 (1.34, 1.40); p<0.001 | | | | 1.33 (1.31, 1.35); p<0.001 | | | |

FGRS_AUD_ = family genetic risk score for alcohol use disorder
