# Supplementary material for: Socioeconomic position indicators and risk of alcohol-related medical conditions: A national cohort study from Sweden
Source: PLoS Med. 2024 Mar 19;21(3):e1004359. doi: 10.1371/journal.pmed.1004359 (PMC10950249; doi:10.1371/journal.pmed.1004359)
Supplement: S12 Table — Hazard ratios, 95% confidence intervals, and Chi-square p-values are presented. The primary predictors of interest (education level and income) were modeled using time-varying coefficients, with a linear term for time. Below, we provide snapshots of hazard ratios for education level and income at 4 time points: at the beginning of observation (time 0), after 5 years, after 10 years, and after 15 years. These secondary analyses were limited to the subsample born in Sweden with 2 Swedish-born parents to improve the precision of the family genetic risk score for alcohol use disorder; accordingly, region of interest is excluded as a covariate. (DOCX) [file pmed.1004359.s013.docx]

**S12 Table.** Complete results for Model S3 for females and males, testing the associations between education level and income with alcohol-related medical conditions. Hazard ratios, 95% confidence intervals, and Chi-square p-values are presented. The primary predictors of interest (education level and income) were modeled using time-varying coefficients, with a linear term for time. Below, we provide snapshots of hazard ratios for education level and income at four timepoints: at the beginning of observation (time 0), after 5 years, after 10 years, and after 15 years. These secondary analyses were limited to the subsample born in Sweden with two Swedish-born parents to improve the precision of the family genetic risk score for alcohol use disorder; accordingly, region of interest is excluded as a covariate.

|  | *Females* | | | | *Males* | | | |
| --- | --- | --- | --- | --- | --- | --- | --- | --- |
| *Variable* | Time 0 | 5 years | 10 years | 15 years | Time 0 | 5 years | 10 years | 15 years |
| Education  low vs. high | 2.63  (2.06, 3.34); p<0.001 | 2.48 (2.08, 2.95); p<0.001 | 2.34  (2.08, 2.64); p<0.001 | 2.21  (1.99, 2.45); p<0.001 | 1.59  (1.38, 1.82); p<0.001 | 1.53  (1.39, 1.70); p<0.001 | 1.49  (1.38, 1.59); p<0.001 | 1.44  (1.35, 1.53); p<0.001 |
| Education  mid vs. high | 1.54  (1.27, 1.89); p<0.001 | 1.53  (1.33, 1.77); p<0.001 | 1.52  (1.37, 1.67); p<0.001 | 1.50  (1.38, 1.64); p<0.001 | 1.23  (1.09, 1.38); p<0.001 | 1.22  (1.12, 1.33); p<0.001 | 1.22  (1.15, 1.29); p<0.001 | 1.21  (1.15, 1.29); p<0.001 |
| Income quartile  1 vs. 4 | 6.98  (5.41, 9.00); p<0.001 | 5.16  (4.27, 6.24); p<0.001 | 3.82  (3.32, 4.39); p<0.001 | 2.82  (2.50, 3.19); p<0.001 | 6.61  (5.63, 7.76); p<0.001 | 4.77  (4.24, 5.37); p<0.001 | 3.45  (3.16, 3.76); p<0.001 | 2.49  (2.31, 2.68); p<0.001 |
| Income quartile  2 vs. 4 | 2.76  (2.16, 3.58); p<0.001 | 2.24  (1.85, 2.72); p<0.001 | 1.82  (1.59, 2.09); p<0.001 | 1.48  (1.32, 1.66); p<0.001 | 2.34  (1.97, 2.78); p<0.001 | 1.94  (1.71, 2.19); p<0.001 | 1.60  (1.46, 1.75); p<0.001 | 1.32  (1.23, 1.43; p<0.001) |
| Income quartile  3 vs. 4 | 1.29  (0.98, 1.69); p=0.068 | 1.20  (0.99, 1.47); p=0.072 | 1.12  (0.98, 1.28); p=0.109 | 1.04  (0.94, 1.16); p=0.453 | 1.35  (1.13, 1.60); p<0.001 | 1.23  (1.08, 1.39); p=0.002 | 1.12  (1.01, 1.22); p=0.013 | 1.02  (0.95, 1.09); p=0.578 |
| Birth year | 1.02 (1.01, 1.03); p<0.001 | | | | 1.01 (1.00, 1.01); p=0.002 | | | |
| Marital status |  | | | |  | | | |
| Married | Reference | | | | Reference | | | |
| Unmarried | 0.90 (0.82, 0.99); p=0.035 | | | | 1.29 (1.21, 1.36); p<0.001 | | | |
| Divorced | 1.30 (1.17, 1.44); p<0.001 | | | | 1.60 (1.49, 1.72); p<0.001 | | | |
| Widowed | 1.37 (0.95, 1.98); p=0.089 | | | | 1.99 (1.36, 2.90); p<0.001 | | | |
| FGRS_AUD_ | 1.31 (1.28, 1.34); p<0.001 | | | | 1.29 (1.27, 1.31); p<0.001 | | | |
| Internalizing disorders | 2.54 (2.37, 2.73); p<0.001 | | | | 3.10 (2.96, 3.24); p<0.001 | | | |
| Externalizing disorders | 3.48 (3.14, 3.85); p<0.001 | | | | 2.23 (2.08, 2.39) | | | |

FGRS_AUD_ = family genetic risk score for alcohol use disorder
