# Supplementary material for: Socioeconomic position indicators and risk of alcohol-related medical conditions: A national cohort study from Sweden
Source: PLoS Med. 2024 Mar 19;21(3):e1004359. doi: 10.1371/journal.pmed.1004359 (PMC10950249; doi:10.1371/journal.pmed.1004359)
Supplement: S13 Table — Hazard ratios, 95% confidence intervals, and Cho-square p-values are presented. The primary predictors of interest (education level and income) were modeled using time-varying coefficients, with a linear term for time. Below, we provide snapshots of hazard ratios for education level and income at 4 time points: at the beginning of observation (time 0), after 5 years, after 10 years, and after 15 years. These secondary analyses were limited to the subsample born in Sweden with 2 Swedish-born parents to improve the precision of the family genetic risk score for alcohol use disorder; accordingly, region of interest is excluded as a covariate. (DOCX) [file pmed.1004359.s014.docx]

**S13 Table.** Complete results for Model S4 for females and males, testing the associations between education level and income with alcohol-related medical conditions. Hazard ratios, 95% confidence intervals, and Cho-square p-values are presented. The primary predictors of interest (education level and income) were modeled using time-varying coefficients, with a linear term for time. Below, we provide snapshots of hazard ratios for education level and income at four timepoints: at the beginning of observation (time 0), after 5 years, after 10 years, and after 15 years. These secondary analyses were limited to the subsample born in Sweden with two Swedish-born parents to improve the precision of the family genetic risk score for alcohol use disorder; accordingly, region of interest is excluded as a covariate.

|  | *Females* | | | | *Males* | | | |
| --- | --- | --- | --- | --- | --- | --- | --- | --- |
| *Variable* | Time 0 | 5 years | 10 years | 15 years | Time 0 | 5 years | 10 years | 15 years |
| Education  low vs. high | 2.10  (1.65, 2.68); p<0.001 | 2.03  (1.71, 2.42); p<0.001 | 1.96  (1.74, 2.22); p<0.001 | 1.90  (1.71, 2.11); p<0.001 | 1.46  (1.27, 1.68); p<0.001 | 1.42  (1.28, 1.57); p<0.001 | 1.37  (1.28, 1.47); p<0.001 | 1.33  (1.25, 1.41); p<0.001 |
| Education  mid vs. high | 1.39  (1.14, 1.69); p=0.001 | 1.39  (1.20, 1.60); p<0.001 | 1.38  (1.25, 1.53); p<0.001 | 1.38  (1.27, 1.50); p<0.001 | 1.15  (1.02, 1.30); p=0.020 | 1.15  (1.05, 1.25); p=0.002 | 1.14  (1.07, 1.21); p<0.001 | 1.13  (1.07, 1.19); p<0.001 |
| Income quartile  1 vs. 4 | 5.40  (4.19, 6.97); p<0.001 | 4.10  (3.39, 4.96); p<0.001 | 3.12  (2.71, 3.58); p<0.001 | 2.37  (2.09, 2.67); p<0.001 | 5.61  (4.78, 6.57); p<0.001 | 4.08  (3.63, 4.60); p<0.001 | 2.97  (2.73, 3.24); p<0.001 | 2.17  (2.01, 2.33); p<0.001 |
| Income quartile  2 vs. 4 | 2.43  (1.87, 3.14); p<0.001 | 2.01  (1.66, 2.43); p<0.001 | 1.66  (1.45, 1.91); p<0.001 | 1.38  (1.22, 1.49); p<0.001 | 2.17  (1.83, 2.58); p<0.001 | 1.81  (1.60, 2.06); p<0.001 | 1.51  (1.39, 1.66); p<0.001 | 1.26  (1.17, 1.36); p<0.001 |
| Income quartile  3 vs. 4 | 1.30  (0.99, 1.72); p=0.061 | 1.22  (1.00, 1.49); p=0.047 | 1.15  (1.01, 1.32); p=0.041 | 1.09  (0.98, 1.21); p=0.125 | 1.32  (1.11, 1.58); p=0.002 | 1.22  (1.07, 1.38); p=0.002 | 1.12  (1.03, 1.22); p=0.011 | 1.03  (0.96, 1.11); p=0.374 |
| Birth year | 1.03 (1.02, 1.04); p<0.001 | | | | 1.02 (1.02, 1.03); p<0.001 | | | |
| Marital status |  | | | |  | | | |
| Married | Reference | | | | Reference | | | |
| Unmarried | 0.87 (0.79, 0.96); p=0.008 | | | | 1.23 (1.16, 1.30); p<0.001 | | | |
| Divorced | 1.09 (0.98, 1.21); p=0.130 | | | | 1.40 (1.30, 1.51); p<0.001 | | | |
| Widowed | 1.15 (0.80, 1.66); p=0.449 | | | | 1.85 (1.26, 2.70); p=0.002 | | | |
| FGRS_AUD_ | 1.17 (1.15, 1.20); p<0.001 | | | | 1.21 (1.19, 1.23); p<0.001 | | | |
| Internalizing disorders | 1.26 (1.17, 1.36); p<0.001 | | | | 1.80 (1.71, 1.88); p<0.001 | | | |
| Externalizing disorders | 1.25 (1.13, 1.39); p<0.001 | | | | 1.28 (1.19, 1.37); p<0.001 | | | |
| AUD | 31.35 (28.94, 33.96); p<0.001 | | | | 10.70 (10.22, 11.22); p<0.001 | | | |

FGRS_AUD_ = family genetic risk score for alcohol use disorder; AUD=alcohol use disorder
