# Supplementary material for: Socioeconomic position indicators and risk of alcohol-related medical conditions: A national cohort study from Sweden
Source: PLoS Med. 2024 Mar 19;21(3):e1004359. doi: 10.1371/journal.pmed.1004359 (PMC10950249; doi:10.1371/journal.pmed.1004359)
Supplement: S14 Table — Hazard ratios, 95% confidence intervals, and Chi-square p-values are presented. The primary predictors of interest (education level and income) were modeled using time-varying coefficients, with a linear term for time. Below, we provide snapshots of hazard ratios for education level and income at 4 time points: at the beginning of observation (time 0), after 5 years, after 10 years, and after 15 years. These secondary analyses were limited to the subsample born in Sweden with 2 Swedish-born parents to improve the precision of the family genetic risk score for alcohol use disorder; accordingly, region of interest is excluded as a covariate. This model includes an interaction term between FGRSAUD and each level of education and income; those results are in S15 Table. (DOCX) [file pmed.1004359.s015.docx]

**S14 Table.** Complete results for Model S5 for females and males, testing the associations between education level and income with alcohol-related medical conditions. Hazard ratios, 95% confidence intervals, and Chi-square p-values are presented. The primary predictors of interest (education level and income) were modeled using time-varying coefficients, with a linear term for time. Below, we provide snapshots of hazard ratios for education level and income at four timepoints: at the beginning of observation (time 0), after 5 years, after 10 years, and after 15 years. These secondary analyses were limited to the subsample born in Sweden with two Swedish-born parents to improve the precision of the family genetic risk score for alcohol use disorder; accordingly, region of interest is excluded as a covariate. This model includes an interaction term between FGRS_AUD_ and each level of education and income; those results are in Supplementary Table S15.

|  | *Females* | | | | *Males* | | | |
| --- | --- | --- | --- | --- | --- | --- | --- | --- |
| *Variable* | Time 0 | 5 years | 10 years | 15 years | Time 0 | 5 years | 10 years | 15 years |
| Education  low vs. high | 2.24  (1.74, 2.89); p<0.001 | 2.16  (1.79, 2.62); p<0.001 | 2.08  (1.81, 2.40); p<0.001 | 2.01  (1.77, 2.28); p<0.001 | 1.55  (1.34, 1.79); p<0.001 | 1.50  (1.34, 1.67); p<0.001 | 1.45  (1.34, 1.57); p<0.001 | 1.40  (1.30, 1.51); p<0.001 |
| Education  mid vs. high | 1.48  (1.21, 1.83); p<0.001 | 1.48  (1.27, 1.73); p<0.001 | 1.47  (1.32, 1.65); p<0.001 | 1.47  (1.33, 1.62); p<0.001 | 1.20  (1.06, 1.36); p<0.001 | 1.19  (1.09, 1.31); p<0.001 | 1.18  (1.11, 1.27); p<0.001 | 1.18  (1.11, 1.25); p<0.001 |
| Income quartile  1 vs. 4 | 5.41  (4.16, 7.05); p<0.001 | 4.12  (3.37, 5.04); p<0.001 | 3.13  (2.68, 3.66); p<0.001 | 2.38  (2.07, 2.74); p<0.001 | 5.69  (4.83, 6.71); p<0.001 | 4.14  (3.66, 4.69); p<0.001 | 3.02  (2.74, 3.31); p<0.001 | 2.20  (2.02, 2.39); p<0.001 |
| Income quartile  2 vs. 4 | 2.52  (1.93, 3.30); p<0.001 | 2.09  (1.71, 2.69); p<0.001 | 1.73  (1.49, 2.02); p<0.001 | 1.44  (1.26, 1.64); p<0.001 | 1.79  (1.57, 2.04); p<0.001 | 1.79  (1.57, 2.04); p<0.001 | 1.50  (1.36, 1.65); p<0.001 | 1.25  (1.15, 1.36); p<0.001 |
| Income quartile  3 vs. 4 | 1.32  (1.00, 1.75); p=0.052 | 1.25  (1.01, 1.54); p=0.039 | 1.18  (1.01, 1.37); p=0.036 | 1.11  (0.98, 1.26); p=0.104 | 1.28  (1.07, 1.54); p=0.006 | 1.18  (1.03, 1.35); p=0.014 | 1.09  (0.99, 1.20); p=0.088 | 1.00  (0.92, 1.08); 0.969 |
| Birth year | 1.03 (1.02, 1.04); p<0.001 | | | | 1.02 (1.02, 1.03); p<0.001 | | | |
| Marital status |  | | | |  | | | |
| Married | Reference | | | | Reference | | | |
| Unmarried | 0.88 (0.79, 0.97); p=0.008 | | | | 1.23 (1.16, 1.30); p<0.001 | | | |
| Divorced | 1.09 (0.98, 1.21); p=0.126 | | | | 1.40 (1.30, 1.51); p<0.001 | | | |
| Widowed | 1.15 (0.80, 1.66); p=0.447 | | | | 1.88 (1.28, 2.75); p=0.001 | | | |
| FGRS_AUD_ | 1.26 (1.18, 1.33); p<0.001 | | | | 1.25 (1.20, 1.30); p<0.001 | | | |
| Internalizing disorders | 1.26 (1.17, 1.36); p<0.001 | | | | 1.80 (1.71, 1.88); p<0.001 | | | |
| Externalizing disorders | 1.25 (1.13, 1.39); p<0.001 | | | | 1.28 (1.19, 1.37); p<0.001 | | | |
| AUD | 31.31 (28.91, 33.92); p<0.001 | | | | 10.68 (10.02, 11.20); p<0.001 | | | |

FGRS_AUD_ = family genetic risk score for alcohol use disorder; AUD=alcohol use disorder
