# Supplementary material for: Socioeconomic position indicators and risk of alcohol-related medical conditions: A national cohort study from Sweden
Source: PLoS Med. 2024 Mar 19;21(3):e1004359. doi: 10.1371/journal.pmed.1004359 (PMC10950249; doi:10.1371/journal.pmed.1004359)
Supplement: S15 Table — We present these as interactions on the additive scale through the use of the relative excess risk due to interaction (RERI) and synergy index (S), along with corresponding 95% confidence intervals; the p-value is based on a Chi-square test of the interaction term. These terms are estimated based on the effect of education and income at time 0. (DOCX) [file pmed.1004359.s016.docx]

**S15 Table.** Model S5 includes an interaction term between FGRS_AUD_ and each level of education and income (main effects are provided in Supplementary Table 11). We present these as interactions on the additive scale through the use of the Relative Excess Risk due to Interaction (RERI) and Synergy Index (S), along with corresponding 95% confidence intervals; the p-value is based on a Chi-square test of the interaction term. These terms are estimated based on the effect of education and income at time 0.

|  | **RERI** | **S** | **p-value** |
| --- | --- | --- | --- |
| **Females** |  |  |  |
| Low vs high education × FGRS_AUD_ | 0.15 (-0.01, 0.31) | 1.11 (1.01, 1.21) | 0.068 |
| Mid vs high education × FGRS_AUD_ | 0.00 (-0.09, 0.09) | 1.00 (0.88, 1.13) | 0.994 |
| Income quartile 1 vs 4 × FGRS_AUD_ | 1.02 (0.59, 1.46) | 1.22 (1.15, 1.29) | <0.001 |
| Income quartile 2 vs 4 × FGRS_AUD_ | 0.25 (0.06, 0.44) | 1.14 (1.06, 1.24) | 0.010 |
| Income quartile 3 vs 4 × FGRS_AUD_ | 0.04 (-0.09, 0.16) | 1.07 (0.88, 1.29) | 0.559 |
|  |  |  |  |
| **Males** |  |  |  |
| Low vs high education × FGRS_AUD_ | 0.03 (-0.04, 0.10) | 1.04 (0.95, 1.13) | 0.410 |
| Mid vs high education × FGRS_AUD_ | -0.02 (-0.06, 0.03) | 0.96 (0.85, 1.09) | 0.541 |
| Income quartile 1 vs 4 × FGRS_AUD_ | 1.06 (0.78, 1.33) | 1.22 (1.18, 1.26) | <0.001 |
| Income quartile 2 vs 4 × FGRS_AUD_ | 0.32 (0.19, 0.45) | 1.23 (1.16, 1.30) | <0.001 |
| Income quartile 3 vs 4 × FGRS_AUD_ | 0.13 (0.03, 0.22) | 1.23 (1.10, 1.38) | <0.007 |

FGRS_AUD_ = family genetic risk score for alcohol use disorder
