# Supplementary material for: Socioeconomic position indicators and risk of alcohol-related medical conditions: A national cohort study from Sweden
Source: PLoS Med. 2024 Mar 19;21(3):e1004359. doi: 10.1371/journal.pmed.1004359 (PMC10950249; doi:10.1371/journal.pmed.1004359)
Supplement: S1 STROBE Checklist — (DOCX) [file pmed.1004359.s017.docx]

STROBE Statement—checklist of items that should be included in reports of observational studies

|  | Item No. | Recommendation | Page  No. | Relevant text from manuscript |
| --- | --- | --- | --- | --- |
| **Title and abstract** | 1 | (*a*) Indicate the study’s design with a commonly used term in the title or the abstract | Title page |  |
|  |  | (*b*) Provide in the abstract an informative and balanced summary of what was done and what was found | Abstract |  |
| Introduction | | | |  |
| Background/rationale | 2 | Explain the scientific background and rationale for the investigation being reported | Introduction; paragraphs 2-4 | Extensive |
| Objectives | 3 | State specific objectives, including any prespecified hypotheses | Introduction; paragraph 4 | “We hypothesized that lower SEP indicators would be associated with higher risk of AMC, and that these associations would be attenuated but persist after accounting for covariates and potential confounders. |
| Methods | | | |  |
| Study design | 4 | Present key elements of study design early in the paper | Introduction, paragraph 4; | “In the current study, we aimed to clarify the association between SEP and AMC through the use of longitudinal, nationwide Swedish registry data and Cox proportional hazards models.” |
| Setting | 5 | Describe the setting, locations, and relevant dates, including periods of recruitment, exposure, follow-up, and data collection | Materials and Methods, paragraphs 1, 2, 3 | “We used several Swedish nationwide registers…”  “we included females and males, born between 1950 and 1970, and residing in Sweden at age 40, without a prior AMC registration.”  “Our outcome variable, AMC, was defined from Swedish medical registers by the following ICD codes…”  “The primary independent variables of interest were education and familial income…” |
| Participants | 6 | (*a*) *Cohort study*—Give the eligibility criteria, and the sources and methods of selection of participants. Describe methods of follow-up  *Case-control study*—Give the eligibility criteria, and the sources and methods of case ascertainment and control selection. Give the rationale for the choice of cases and controls  *Cross-sectional study*—Give the eligibility criteria, and the sources and methods of selection of participants | Materials and Methods, paragraphs 1, 8 | "we included females and males, born between 1950 and 1970, and residing in Sweden at age 40, without a prior AMC registration”  “Cox proportional hazard models were utilized to estimate the time to AMC, censoring at end of follow-up (December 2018), death, or emigration…” |
|  |  | (*b*) *Cohort study*—For matched studies, give matching criteria and number of exposed and unexposed  *Case-control study*—For matched studies, give matching criteria and the number of controls per case | NA |  |
| Variables | 7 | Clearly define all outcomes, exposures, predictors, potential confounders, and effect modifiers. Give diagnostic criteria, if applicable | Materials and Methods, paragraphs 2-7 | Extensive; “Our outcome variable, AMC, was defined from Swedish medical registers by the following ICD codes…”; “The primary independent variables of interest were education and familial income. Income was assessed at age 40 and categorized based on the income quartiles for the working population…”; “Sociodemographic covariates included year of birth, marital status, and region of origin”; “From Swedish medical registers, AUD was defined by the following ICD codes…”; “Internalizing disorder (ID) was defined using the ICD codes…”; “Externalizing disorder (ED) was defined…”; “Finally, aggregate genetic liability for AUD, which was used in a series of secondary analyses, was operationalized…” |
| Data sources/ measurement | 8* | For each variable of interest, give sources of data and details of methods of assessment (measurement). Describe comparability of assessment methods if there is more than one group | Materials and Methods; paragraphs 2-7 | Same as above |
| Bias | 9 | Describe any efforts to address potential sources of bias | NA | NB: These are population-wide registers, greatly minimizing bias concerns. |
| Study size | 10 | Explain how the study size was arrived at | Materials and Methods, paragraph 2 | “we included females and males, born between 1950 and 1970, and residing in Sweden at age 40, without a prior AMC registration…” |

| Quantitative variables | 11 | Explain how quantitative variables were handled in the analyses. If applicable, describe which groupings were chosen and why | Materials and Methods, Statistical Analyses, paragraph 2 | “We then pursued model building to estimate the association between education and/or income with AMC…” |
| --- | --- | --- | --- | --- |
| Statistical methods | 12 | (*a*) Describe all statistical methods, including those used to control for confounding | Materials and Methods, Statistical analyses, paragraphs 2, 3, 4, 5 | “We then pursued model building to estimate the association between education and/or income with AMC…” |
|  |  | (*b*) Describe any methods used to examine subgroups and interactions | Materials and Methods, Statistical analyses, paragraphs 1, 4, 5 | “In a secondary set of analyses, we included genetic risk of AUD…”;  “Models were stratified by sex to facilitate direct comparisons and enhance data transparency.”  “We ran an additional model, Model S5, including an interaction between FGRSAUD and education and between FGRSAUD and income, to test whether genetic liability to AUD moderated the associations between education/income and AMC…” |
|  |  | (*c*) Explain how missing data were addressed | Materials and Methods, Sample, paragraph 1 | “Due to missing data on education, N=916 females and N=845 males from the cohort were excluded…” |
|  |  | (*d*) *Cohort study*—If applicable, explain how loss to follow-up was addressed  *Case-control study*—If applicable, explain how matching of cases and controls was addressed  *Cross-sectional study*—If applicable, describe analytical methods taking account of sampling strategy | Materials and Methods, Statistical analyses, paragraph 1 | “Cox proportional hazard models were utilized to estimate the time to AMC, censoring at end of follow-up (December 2018), death, or emigration.” |
|  |  | (*e*) Describe any sensitivity analyses | NA |  |
| Results | | | | |
| Participants | 13* | (a) Report numbers of individuals at each stage of study—eg numbers potentially eligible, examined for eligibility, confirmed eligible, included in the study, completing follow-up, and analysed | Tables 1-2;  Materials and Methods, Sample, paragraph 1 | “Due to missing data on education, N=916 females and N=845 males from the cohort were excluded…”; Ns provided in Tables 1-2 |
|  |  | (b) Give reasons for non-participation at each stage | NA |  |
|  |  | (c) Consider use of a flow diagram | NA |  |
| Descriptive data | 14* | (a) Give characteristics of study participants (eg demographic, clinical, social) and information on exposures and potential confounders | p9- p10, p26-p27 | Table 1 and 2 |
|  |  | (b) Indicate number of participants with missing data for each variable of interest | Materials and Methods, Sample | “Due to missing data on education, N=916 females and N=845 males from the cohort were excluded…” |
|  |  | (c) *Cohort study*—Summarise follow-up time (eg, average and total amount) | Tables 1 and 2 | Mean and range of follow-up times provided in Tables 1-2 |
| Outcome data | 15* | *Cohort study*—Report numbers of outcome events or summary measures over time | Tables 1 and 2 | Table 1 and 2 |
|  |  | *Case-control study—*Report numbers in each exposure category, or summary measures of exposure | *NA* |  |
|  |  | *Cross-sectional study—*Report numbers of outcome events or summary measures | *NA* |  |
| Main results | 16 | (*a*) Give unadjusted estimates and, if applicable, confounder-adjusted estimates and their precision (eg, 95% confidence interval). Make clear which confounders were adjusted for and why they were included | Results, Crude Models, paragraph 1; Results, Multivariable models, paragraphs 1-5 | Figure 1, Table S4-S15;  Extensive text throughout Results section |
|  |  | (*b*) Report category boundaries when continuous variables were categorized | Materials and Methods, Measures, paragraph 2 | “The primary independent variables of interest were education and familial income. Income was assessed at age 40 and categorized based on the income quartiles for the working population, aged 20 to 65, in Sweden, but analyses included both employed and unemployed individuals. Educational attainment was categorized into low (compulsory school only), mid (upper secondary school), and high (university level).” |
|  |  | (*c*) If relevant, consider translating estimates of relative risk into absolute risk for a meaningful time period | Supplementary Material | Table S1 |

Continued on next page

| Other analyses | 17 | Report other analyses done—eg analyses of subgroups and interactions, and sensitivity analyses | Results, Secondary analyses, paragraph 1 | “We pursued a secondary set of analyses that corresponded to the original models, replacing region of origin with FGRSAUD, as described in the Methods…” |
| --- | --- | --- | --- | --- |
| Discussion | | | | |
| Key results | 18 | Summarise key results with reference to study objectives | Discussion, paragraph 1 | “Even after accounting for other sociodemographic measures, psychopathology, and the role of AUD itself, individuals with lower levels of education and income had an increased risk of AMC…” |
| Limitations | 19 | Discuss limitations of the study, taking into account sources of potential bias or imprecision. Discuss both direction and magnitude of any potential bias | Discussion, paragraphs 9, 10 | “Our findings must still be viewed in the context of several limitations…” |
| Interpretation | 20 | Give a cautious overall interpretation of results considering objectives, limitations, multiplicity of analyses, results from similar studies, and other relevant evidence | Discussion, paragraph 11 | “In summary, we provide evidence that individuals with lower levels of education and/or income are more likely to suffer from AMC, even after accounting for differences in AUD, comorbid psychopathology, and aggregate genetic liability…” |
| Generalisability | 21 | Discuss the generalisability (external validity) of the study results | Discussion, paragraph 10 | “Finally, alcohol consumption varies across countries and cultures, and the current findings might not generalize to other contexts…” |
| Other information | |  | | |
| Funding | 22 | Give the source of funding and the role of the funders for the present study and, if applicable, for the original study on which the present article is based | Title page | “Funding: This project was supported by grant AA023534 from the US National Institutes of Health, and grants from the Swedish Research Council to Jan Sundquist (2020-01175) as well as ALF funding from Region Skåne awarded to Kristina Sundquist.” |

*Give information separately for cases and controls in case-control studies and, if applicable, for exposed and unexposed groups in cohort and cross-sectional studies.

**Note:** An Explanation and Elaboration article discusses each checklist item and gives methodological background and published examples of transparent reporting. The STROBE checklist is best used in conjunction with this article (freely available on the Web sites of PLoS Medicine at http://www.plosmedicine.org/, Annals of Internal Medicine at http://www.annals.org/, and Epidemiology at http://www.epidem.com/). Information on the STROBE Initiative is available at www.strobe-statement.org.
