# Supplementary material for: Socioeconomic position indicators and risk of alcohol-related medical conditions: A national cohort study from Sweden
Source: PLoS Med. 2024 Mar 19;21(3):e1004359. doi: 10.1371/journal.pmed.1004359 (PMC10950249; doi:10.1371/journal.pmed.1004359)
Supplement: Attachment — Submitted filename: edwards.pdf [file pmed.1004359.s018.pdf]

This paper uses a number of Swedish databases to examine associations with alcohol–related medical conditions (AMC).

I was asked for a statistical report and I interpret that to include all aspects of the design and conduct of the study. The version I received did not have page numbers. My numbering has the abstract starting on page 2

Points of detail

Page 2 I do not understand what the phrase 'across low/mid education levels and year of observation' means.

Page 6 Somewhere about here in the methods I think a sentence justifying the choice to use two cohorts, one for men and one for women, would be helpful. I am not querying the decision but making the reasoning explicit would be good practice.

Page 6 Categorising an essentially continuous variable wastes information (Altman and Royston, 2006; Royston et al., 2006) and leads to models which are often implausible as they predict the effect remaining flat within categories and then jumping to a new value at the category boundary. So why was income categorised and why these categories?

Page 6 Presumably region of origin should have a category for Sweden? Or are they included in Western Countries? Tables 1 and 2 suggest they were treated separately.

Page 7 Does the sentence starting 'Most of these' imply that most of the crimes included in those categories are in fact drink driving or does it mean that most of the people in the cohort with AUD were drink drivers (in which case it is, strictly speaking, part of the results)? (In my dialect colloquially we call it drink driving but I assume drunk is not a typo. The actual offence has some more complicated name.)

Page 8 Restricting the genetic risk analysis to Swedish born participants is clearly the lesser of two evils here.

Page 9 Some regions seem to be less at risk than Swedes which could be pointed out here for the benefit of people who do not read the table. It is picked up on Page 15 of course.

Page 9 As well as giving us the measures of association between income and education I think it would be very helpful to give us cross–tabulations. They could also include the number of cases of AMC. in each category.

Page 10 Collapsed seems the wrong word here. It made me thing they had been averaged in some way. What I think the authors mean is that in the left–most panels two different models are presented as opposed to the other six panels which each present a single model The word superimposed occurs to me as a possible alternative to collapsed.

Page 11 The phrase 'and for those in higher income quartiles' does not make sense as the rest of the sentence is about the lowest category and change over time. It probably needs splitting off into a separate sentence.

Page 11 Is it surprising that AUD moderates or mediates the effect? It seems difficult to acquire some of the diagnoses in the supplement without sustained problematic drinking.

Page 14 I have a vague memory that similar interactions between sex and marital status had been observed: marriage makes men happy and women unhappy. Or is that just an urban legend?

Figure The lines seem very faint in the PDF I received. I suggest making them thicker.

Supplementary Tables I suggest including a line in the tables for the reference category would help to see where one categorical covariate ends and another begins. Not necessary for the covariates with only two levels.

Point of more substance

I wonder whether for an international readership we need more detail about the epidemiological context and its possible effect on generalisability. For instance, is it necessary to point out that, by European standards, alcohol in Sweden is expensive? Does that have any bearing on conclusions about income level? Systembolaget might be worth a mention too. I am not suggesting this negatively affects the study relevance worldwide.

Summary

Mostly requests for clarification and for more detail.

Michael Dewey

References

D G Altman and P Royston. The cost of dichotomising continuous variables. *British Medical Journal*, 332:1080, 2006.

P Royston, D G Altman, and W Sauerbrei. Dichotomizing continuous predictors in multiple regression: a bad idea. *Statistics in Medicine*, 25:127–141, 2006.
