# Supplementary material for: Socioeconomic position indicators and risk of alcohol-related medical conditions: A national cohort study from Sweden
Source: PLoS Med. 2024 Mar 19;21(3):e1004359. doi: 10.1371/journal.pmed.1004359 (PMC10950249; doi:10.1371/journal.pmed.1004359)
Supplement: Attachment — Submitted filename: responses-to-reviewers-2024-01-29.pdf [file pmed.1004359.s019.pdf]


COMMENTS FROM THE ACADEMIC EDITOR
A major revision seems reasonable.
Response: We are very grateful for the opportunity to revise and improve upon our initial submission. Please note that, in the course of re-running analyses to address some Reviewer concerns, we identified a coding error. This was corrected and has not resulted in any substantive changes to our hazard ratio estimates or interpretations. However, the Editors and Reviewers will see throughout that most hazard ratios and confidence intervals shifted slightly, resulting in many tracked changes, particularly to Supplementary Tables. We regret this oversight and are happy to provide a “clean” version of the revised documents to improve readability.

I had just two thoughts to add:
1. Maybe I missed this, but why limit the lower end of the age range to 40 years? The listed alcohol-related medical conditions do occur in younger populations. Is it a sample size problem?
Response: This decision was based on our efforts to (i) follow the study subjects from an age when they had reached a somewhat stable level on their socioeconomic variables, and (ii) ensure that we had a sufficient sample size of AMC to avoid imprecise estimates. Because AMC before the age of 40 was quite rare, we selected this age limit.

2. I wonder if the authors might consider making a conceptual model to visually depict the relationships and confounders they include in the models. I understand that it isn't a fully causal DAG, but I find that such figures tend to help readers a lot. This is not a mandatory comment, just a suggestion for interpretability/readability.
Response: We appreciate this suggestion. We have elected not to include a visual depiction of the model for two primary reasons. First, as the Editor notes, this is not a causal model, and we are concerned that readers who focus on the visual depiction of the model might misinterpret it as a DAG. Second, in the models that included $FGRS_{AUD}$, that variable is coded first as a covariate, and then, where appropriate, it is also included in an interaction term. Thus, the statistical models do not always clearly translate to a visual conceptual model. We hope that this is acceptable.

COMMENTS FROM THE EDITORS
GENERAL
1) Please respond to all editor and reviewer comments detailed below, in full.
Response: We have responded to each comment.

2) Please include line and page numbers to aid reviewing and editing.
Response: We have included line and page numbers to orient Reviewers and Editors to our changes.

3) Please ensure that the study is reported according to the STROBE guideline, and include the completed STROBE checklist as Supporting Information. Please add the following statement, or similar, to the Methods: "This study is reported as per the Strengthening the Reporting of Observational Studies in Epidemiology (STROBE) guideline (S1 Checklist)."
The STROBE guideline can be found here: https://urldefense.com/v3/__http://www.equator-network.org/reporting-guidelines/strobe/__;!!JqxBPMk!nhZ1GKdLS_h91yJCRE7K2tC6R9zeol80C04bh49LdzSESCqpNmjRfjoANJ-uShlsXJ2A2qU6-JIxzUTzep3l$

When completing the checklist, please use section and paragraph numbers, rather than page and/or line numbers as these often change in the event of publication.
Response: We have included the STROBE checklist as a supplementary file in this revision, with section and paragraph numbers completed where applicable.

TITLE
4) Please revise your title according to PLOS Medicine's style. Your title must be nondeclarative and not a question. It should begin with main concept if possible. "Effect of" should be used only if causality can be inferred, i.e., for an RCT. Please place the study design ("A randomized controlled trial," "An observational study," "A modelling study," etc.) in the subtitle (ie, after a colon), as in the current version.
Response: We have retitled our submission (“Socioeconomic position indicators and risk of alcohol-related medical conditions: An observational Swedish national cohort study”) to be consistent with the journal’s style.

5) We note and thank the reviewer (please see below) for their interest in the genetic risk score and for the comments regarding its reference in title. We agree that it is very interesting, but as it forms part of your secondary analyses, we don’t think it necessary for the title to specifically detail this or for extensive additional details to be added to the abstract.
Response: We have deferred to the editors on this point. Accordingly, our only change to the abstract with respect to the genetic risk score is to note which registers are used for its derivation (as requested in Editors’ comment 7 below).

ABSTRACT
Abstract Background:
6) Please ensure that the final sentence clearly states the study question.
Response: We have modified the sentence so that it more clearly addresses our research aim.

Abstract Methods and Findings:
7) Please include (brief) details of the registries used to leverage your data including those used to obtain FGRS.
Response: We have now noted that FGRS are estimated using data from medical, criminal, and pharmacy records.

8) Please clearly define the length of follow-up (mean, SD, range).
Response: This information has been added to Tables 1 and 2.

9) Please provide details of the important dependent variables that you refer to.
Response: We have now clarified in the Abstract that alcohol-related medical conditions (the only dependent variable) are based on ICD codes, and include outcomes such as alcoholic hepatitis and alcoholic cardiomyopathy. Because the Abstract is already quite long and we are mindful of addressing as many Editor/Reviewer comments as possible, we have made an effort to keep this content streamlined.

10) Please include the actual amounts and/or absolute risk(s) of relevant outcomes, not only the relative measures (example for absolute risks: PMID: 28399126).
Response: We agree that this information is useful for readers and have added the absolute risks to the Abstract. We now also provide a Supplementary Table (Supplementary Table S1) of incidence rates.

11) Please quantify the main results with 95% CIs and p values. Please report p as <0.001 and where higher the exact p value as p=0.002, for example. Please use commas as opposed to hyphens to separate upper and lower CI bounds as the latter can be confused with reporting of negative values.
Response: We have included 95% CIs and p-values and formatted as requested by the Reviewer in this revision.

12) Please ensure that all abbreviations used for statistical reporting have been defined at first use for the reader.
'…even after adjusting for all other covariates and potential confounders…' as above please provide details of the important dependent variables adjusted for.
Response: All abbreviations are now defined at first use, and the dependent variable (risk of AMC) has been further described.

13) We don't think that it is necessary to present both crude and adjusted analyses in the abstract (but it certainly doesn't hurt to do so) so, if necessary, to conserve space during your revisions you could present only the adjusted results (and the factors adjusted for).
Please define 'AUD' for the reader at first use.
Response: We have defined AUD in this revised version. We appreciate the Editors' flexibility regarding estimates from crude models, and have only provided results from adjusted analyses in this revision.

14) AUTHOR SUMMARY
At this stage, we ask that you include a short, non-technical Author Summary of your research to make findings accessible to a wide audience that includes both scientists and non-scientists. The authors summary should consist of 2-3 succinct bullet points under each of the following headings:
• Why Was This Study Done? Authors should reflect on what was known about the topic before the research was published and why the research was needed.
• What Did the Researchers Do and Find? Authors should briefly describe the study design that was used and the study's major findings. Do include the headline numbers from the study, such as the sample size and key findings.
• What Do These Findings Mean? Authors should reflect on the new knowledge generated by the research and the implications for practice, research, policy, or public health. Authors should also consider how the interpretation of the study's findings may be affected by the study limitations. In the final bullet point of 'What Do These Findings Mean?', please describe the main limitations of the study in non-technical language.
The Author Summary should immediately follow the Abstract in your revised manuscript. This text is subject to editorial change and should be distinct from the scientific abstract. Please see our author guidelines for more information:
https://urldefense.com/v3/__https://journals.plos.org/plosmedicine/s/revising-your-manuscript*loc-author-summary__;Iw!!JqxBPMk!nhZ1GKdLS_h91yJCRE7K2tC6R9zeoI80C04bh49LdzSESCqpNmjRfjoANJ-uShlsXJ2A2qU6-JIxzRBgwjjB$
Response: We have included an Author Summary in this revised manuscript.

INTRODUCTION
15) Final paragraph – suggest revising/moving the sentence beginning 'Our approach…' which seems better suited to the discussion as written. Reference to stratification by sex should remain in the introduction.

Response: We have made a slight modification to this sentence, but believe it is important to inform the readers at this stage about how we envisioned our study making a contribution to this area of research.

16) Penultimate sentence, suggest, ‘We account for a range…’
Response: We have made this change to the text.

METHODS and RESULTS
17) Please also see reviewer comments (below) regarding your methodological approach.
As above, please add the following statement, or similar, to the Methods: "This study is reported as per the Strengthening the Reporting of Observational Studies in Epidemiology (STROBE) guideline (S1 Checklist)."
When completing the checklist, please use section and paragraph numbers, rather than page or line numbers as these often change in the event of publication.
Response: We have included the STROBE checklist as a supplementary file in this revision.

18) Please also include the ethics statement, as requested above, including the name(s) of the institutional review board(s) that provided ethical approval.
Response: We have added this information to the Sample subsection of the Materials and Methods.

19) Did your study have a prospective protocol or analysis plan? Please state this (either way) early in the Methods section.
a) If a prospective analysis plan (from your funding proposal, IRB or other ethics committee submission, study protocol, or other planning document written before analyzing the data) was used in designing the study, please include the relevant prospectively written document with your revised manuscript as a Supporting Information file to be published alongside your study, and cite it in the Methods section. A legend for this file should be included at the end of your manuscript.
b) If no such document exists, please make sure that the Methods section transparently describes when analyses were planned, and when/why any data-driven changes to analyses took place.
c) In either case, changes in the analysis – including those made in response to peer review comments – should be identified as such in the Methods section of the paper, with rationale.
Response: We did not have a formal prospective protocol with a specific analysis plan. We have now noted this in the Statistical Analysis section of the Methods (page 9, line 17). We have also added text to the and in the Supplementary Methods document (in the interest of avoiding an excessively long primary text) to describe the timing of analyses. As noted in our response to the comment below, although we did not have a formal prospective protocol, the only data-driven aspects of our analyses were (i) the determination of birth cohorts and observation periods enabled us to best address our research questions and maximize sample size and follow-up time; (ii) to aid selection of the most appropriate way to model time (e.g., linearly); and (iii) to determine which “snapshot” timepoints of effect sizes would be most informative to readers.

20) For all observational studies, in the manuscript text, please ensure that you indicate: the specific hypotheses you intended to test, the analytical methods by which you planned to test them, the analyses you actually performed, and when reported analyses differ from those that were planned, transparent explanations for differences that affect the reliability of the study's

results. If a reported analysis was performed based on an interesting but unanticipated pattern in the data, please be clear that the analysis was data-driven.
Response: In this revision, we now clearly indicate our hypotheses (page 6, line 19), as well as the methods used and analyses performed (page 6, line 12). As noted above, we did not have a formal prospective analysis plan, and elements of our study design were shaped by preliminary examination of the data. However, we did not conduct analyses other than those that we initially set out to conduct. Where we present “incidental” findings (e.g., of sex differences in the effect of a covariate, marital status), those were observed within the context of our planned analyses; furthermore, those findings are now clearly described as incidental in the text.

21) Results, opening paragraph –
a) please explicitly state the total number of participants included in the full dataset.
Response: We have now added the total N to this paragraph (page 11, line 3).

b) Please clearly define the length of follow up (eg, in mean, SD, and range).
Response: These details are provided in Tables 1 and 2; we have not included them in the text to avoid duplications.

c) Please ensure that you provide the actual numbers of events for outcomes not just summary statistics or OR/HR.
Response: The numbers of actual outcomes (AMC registrations) are provided in the opening paragraph (“In the female cohort, N=4253 individuals had an AMC registration, corresponding to 0.37% of the sample. Among males, N=11,183 (0.93%) had an AMC registration.” Page 11, line 2). These details are also available in Tables 1 and 2.

d) Please ensure that percentages are quantified with numerators and denominators.
Response: We have now added notes to Tables 1 and 2 to clarify the numerators and denominators for each percentage reported.

e) As above for the abstract, please quantify the main results with 95% CIs and p values. Please report p as <0.001 and where higher the exact p value as p=0.002, for example. Please use commas as opposed to hyphens to separate upper and lower CI bounds as the latter can be confused with reporting of negative values. Suggest reporting statistical information as follows, ‘(HR 4.71; 95% CI [3.85, 5.77]; p</=)’. Please check and amend throughout (including supporting information) to ensure consistency of reporting.
Response: We have included 95% CIs and p-values throughout in this revision. The formatting differs slightly depending on whether the HRs are in parentheses, but in all cases, we provide HRs, CIs, and p-values.

f) Please ensure that all abbreviations used for statistical reporting have been defined at first use for the reader.
Response: We have now ensured that all abbreviations are defined at first mention in this revision.

g) When a p value is given, please also include the statistical test used to determine it.
Response: We note in table headings, notes, or the text what kind of test was used for each p-value.

TABLES

22) Throughout (including the supporting information) please ensure that each table is affiliated to an appropriate caption which clearly explains the table content without the need to refer to the text.
Response: We have added detail to the table captions to ensure that they are interpretable without referring to the text.

23) Please ensure that any abbreviations (including those used for statistical reporting) are clearly defined within the caption (or a footnote).
Response: We have added definitions for abbreviations throughout, and regret this oversight in our initial submission.

24) Please ensure that all numerical values are clearly defined for the reader (including in the supporting information).
Response: To the best of our knowledge, all numerical values have been defined.

25) Please report p as <0.001 and where higher the exact p value.
Response: We have formatted as requested in this revision.

26) For main outcomes measures where 95% CIs are presented please also present p values, reporting p as <0.001 and where higher the exact p value.
Response: We now include p-values and have formatted as requested.

27) Throughout (including the supporting information) please ensure that you indicate whether analyses are adjusted or unadjusted. For the purpose of transparent data reporting, where adjusted analyses are presented please present the unadjusted analyses for comparison and clearly detail the factors adjusted in the caption (or footnote)
Response: Where adjusted analyses are presented, all variables in the model are included in the tables; i.e., there are no variables that we adjusted for that we do not present explicitly. The only unadjusted estimates, from crude associations between education/income level and AMC, are presented in the main text (this was an addition in this revision; page 11, line 19).

28) Tables 1 and 2 – please clearly define ‘col/% SD’ in the column header.
Response: We have revised the column headers, and have provided further explanation in the footnotes to Tables 1 and 2.

FIGURES
29) Throughout (including the supporting information), please ensure that each figure is affiliated to an appropriate caption which clearly explains it content without the need to refer to the text. Please ensure that any abbreviations (including those used for statistical reporting) are clearly defined within the caption (or a footnote).
Please see here for guidelines on submitting and citing figures
https://urldefense.com/v3/__https://journals.plos.org/plosmedicine/s/figures*loc-how-to-submit-figures-and-captions__;Iw!!JqxBPMk!nhZ1GKdLS_h91yJCRE7K2tC6R9zeol80C04bh49LdzSESCqpNmjRfjoANJ-uShlsXJ2A2qU6-JIxzQCOF1Xk$
Please consider avoiding the use of green and/or red to make your figures more accessible to those with color blindness.
Response: In this revision, we have provided a more detailed figure caption so that readers do not need to refer to the text to interpret the figure. The figure does not include green or red.

DISCUSSION
Please also see reviewer comments

REFERENCES
30) For in-text reference callouts please place citations in square parentheses separate by commas. For example, [1,3,6] or [1-3]. Please check and amend throughout all sub-sections of the manuscript and supporting files.
Response: We have now applied the PLOS reference style and this has been corrected. We regret this oversight in our previous submission.

31) In the bibliography please ensure that you list up to but no more than 6 author names followed by et al.
Response: Since we have now applied the PLOS reference style and only the first 6 author names are listed.

32) For all web references please ensure you include an, 'Accessed [date].'
Response: We have updated the reference style to include this information.

33) Journal name abbreviations should be those listed in the National Center for Biotechnology Information (NCBI) databases.
Response: As we have now applied the PLOS reference style, this has been corrected.

SUPPORTING INFORMATION
34) Throughout please ensure all requirements detailed above for tables, figures, statistical reporting and referencing are applied to the supporting information, as relevant.
Please cite your Supporting Information as outlined here:
https://urldefense.com/v3/__https://journals.plos.org/plosmedicine/s/supporting-information__;!!JqxBPMk!nhZ1GKdLS_h91yJCRE7K2tC6R9zeol80C04bh49LdzSESCqpNmjRfjoANJ-uShlsXJ2A2qU6-JIxzQYbJJ45$
In the published article, supporting information files are accessed only through a hyperlink attached to the captions. For this reason, you must list captions at the end of your manuscript file. You may include a caption within the supporting information file itself, as long as that caption is also provided in the manuscript file. Do not submit a separate caption file.
Response: We have now included a list of information included in the supplementary material (along with table descriptions) at the end of our manuscript.

COMMENTS FROM THE REVIEWERS

Reviewer #1
This paper uses a number of Swedish databases to examine associations with Alcohol related medical conditions (AMC). I was asked for a statistical report and I interpreted that to include all aspects of the design and conduct of the study. The version I received did not have page numbers. My numbering has the abstract starting on page 2.
Response: We appreciate the reviewer's constructive feedback on our submission. In this revision, we have added page and line numbers.

Points of detail
1) Page 2 I do not understand what the phrase `across low/mid education levels and year of observation' means.

Response: We agree that this could have been written more clearly. High educational attainment was the reference group, and we estimated HRs for low- and mid-level educational attainment. Because we provide “snapshots” of the effect sizes of education (and income) at different points during follow-up, we felt it would be helpful to report the range of effect sizes across time in the Abstract as well. In the revised Abstract, we have reworded (“for low- and mid-level educational attainment across 0-15 years of observation”) and hope this improves clarity.

2) Page 6 Somewhere about here in the methods I think a sentence justifying the choice to use two cohorts, one for men and one for women, would be helpful. I am not querying the decision but making the reasoning explicit would be good practice.
Response: We regret if there was any confusion, but the birth cohort is the same for both sexes. If the reviewer’s comment is referring only to our decision to stratify by sex (for the same birth cohort), the reasoning is that the NIH encourages disaggregation of data by sex when possible (see guidance below), and we are in the fortunate position of being able to conduct well-powered sex-specific studies. We have added this rationale to the Statistical Analysis section of the Methods (page 9, line 20).
NIH guidance: https://orwh.od.nih.gov/sex-gender/orwh-mission-area-sex-gender-in-research/nih-policy-on-sex-as-biological-variable

3) Page 6 Categorising an essentially continuous variable wastes information (Altman and Royston, 2006; Royston et al., 2006) and leads to models which are often implausible as they predict the effect remaining within categories and then jumping to a new value at the category boundary. So why was income categorised and why these categories?
Response: We acknowledge that when categorizing a continuous variable, some information is lost. Nevertheless, we argue that, in this study, such an approach is preferable. The relationship between income and AMC is not linear (on the log HR scale), meaning we would have to transform or investigate various mathematical relationships for the association with income and AMC. We wanted to avoid this for two reasons. First, it will likely make the interpretation of the association more difficult, and second, we wanted to investigate the proportionality association, and be able to communicate how to interpret a change in the associations over follow-up time, which will be more complicated when a linear association between the continuous variable and the outcome cannot be assumed. We worked with three categories of education that corresponds to distinct levels in the Swedish education system, thereby making our results more interpretable under easily-understood conditions. For income we divided the population into quartiles, which we have previously identified as a reasonable categorization.

4) Page 6 Presumably region of origin should have a category for Sweden? Or are they included in Western Countries? Tables 1 and 2 suggest they were treated separately.
Response: We regret the confusion here. Sweden serves as the reference group for the region of origin variable. We have now added a row to all relevant tables indicating as much. We have also indicated in all relevant tables that, for marital status, “married” was the reference group.

5) Page 7 Does the sentence starting `Most of these' imply that most of the crimes included in those categories are in fact drink driving or does it mean that most of the people in the cohort with AUD were drink drivers (in which case it is, strictly speaking, part of the results)? (In my dialect colloquially we call it drink driving but I assume drunk is not a typo. The actual offence has some more complicated name.)
Response: We regret the lack of clarity. We have modified the text to indicate that these codes refer to driving or boating while intoxicated (sometimes referred to as drunk driving, driving under the influence, or drink driving), and that nearly all (99.7%) of criminal registrations are

related to driving while intoxicated, but that most AUD registrations were not identified from the criminal registry alone (page 8, line 20).

6) Page 8 Restricting the genetic risk analysis to Swedish born participants is clearly the lesser of two evils here.
Response: We concur, and are pleased that we are able to include this unique perspective, if only for a subset of the cohort.

7) Page 9 Some regions seem to be less at risk than Swedes which could be pointed out here for the benefit of people who do not read the table. It is picked up on Page 15 of course.
Response: We have added a sentence to this section (page 11, line 12) to highlight the most pronounced differences.

8) Page 9 As well as giving us the measures of association between income and education I think it would be very helpful to give us cross-tabulations. They could also include the number of cases of AMC in each category.
Response: We have added this information as new Supplementary Tables 1-2.

9) Page 10 Collapsed seems the wrong word here. It made me think they had been averaged in some way. What I think the authors mean is that in the left-most panels two different models are presented as opposed to the other six panels which each present a single model. The word superimposed occurs to me as a possible alternative to collapsed.
Response: We agree that “superimposed” is a more accurate term and have made this change.

10) Page 11 The phrase `and for those in higher income quartiles' does not make sense as the rest of the sentence is about the lowest category and change over time. It probably needs splitting off into a separate sentence.
Response: We agree that the phrasing was confusing. We have split this into two sentences as suggested by the Reviewer.

11) Page 11 Is it surprising that AUD moderates or mediates the effect? It seems difficult to acquire some of the diagnoses in the supplement without sustained problematic drinking.
Response: It was not surprising that AUD mediated the effects of income and education, but we felt it was important to test whether those effects were *fully* mediated by AUD. We agree that it would be difficult to acquire many of the AMC registrations in the absence of AUD. Including AUD was a sort of “sanity check” in part (as expected, its effect size was the largest observed).

12) Page 14 I have a vague memory that similar interactions between sex and marital status had been observed: marriage makes men happy and women unhappy. Or is that just an urban legend?
Response: The reviewer is correct that some evidence supports sex differences in the effects of marriage on various health-related outcomes (see references below for examples), though this is not always the case. Our current findings hint the complexity of the role of sex, but we believe that this is an especially interesting area for future research, particularly using data that has more fine-grained information on alcohol consumption.

Kiecolt-Glaser JK, Newton TL. Marriage and health: His and hers. Psychol Bull. 2001;127:472-503. doi: 10.1037/0033-2909.127.4.472.
Kiecolt-Glaser JK, Wilson SJ. Lovesick: How couples’ relationships influence health. Ann Rev Clin Psych. 2017;13:421-43.

13) Figure The lines seem very faint in the PDF I received. I suggest making them thicker.
Response: We appreciate this suggestion and agree that the lines were too faint in our initial submission. In this revision, the thickness of the lines has been increased.

14) Supplementary Tables I suggest including a line in the tables for the reference category would help to see where one categorical covariate ends and another begins. Not necessary for the covariates with only two levels.
Response: We have now added rows to all supplementary tables to make it clear to readers which variables are categorical with more than two levels.

15) I wonder whether for an international readership we need more detail about the epidemiological context and its possible effect on generalisability. For instance, is it necessary to point out that, by European standards, alcohol in Sweden is expensive? Does that have any bearing on conclusions about income level? Systembolaget might be worth a mention too. I am not suggesting this negatively affects the study relevance worldwide.
Response: We appreciate this point, and have added text to the section on limitations accordingly (page 19, line 19).

Reviewer #2: Thank you for the invitation to review this manuscript. I guess it is because I have performed related research previously. The study assesses the association of socioeconomic position with alcohol-related medical conditions. The interplay between different measures of alcohol, SEP measures and different health outcomes related to alcohol and mortality has been studied extensively. The authors do not try to oversell the importance of the paper, they are sober, and argue that the study is an important contribution to the literature because of how they made use of different information from registry data. I don't how a complete overview of comparable studies, but I am impressed by how the authors have used the different registries to define alcohol use disorder, alcohol-related medical conditions, and other study variables, as well as the family genetic risk score for AUD. I can see that these registries and the different definitions has been used previously by the authors.
The manuscript appears to me as being well written and I haven't found anything that I with confidence can say is wrong, although there are some things I would like the authors to clarify. Perhaps the most important is related to the measure of income and the use risk estimates from the final model for predefined covariates, such as to say something about how the risk of AMC differ according to biological sex and background.
Response: We are grateful for the reviewer's positive response to our submission, and hope that our responses below, in conjunction with our changes to the manuscript, have addressed any issues around clarity.

Comment 1
Quoting: "The primary independent variables of interest were education and familial income. Income was assessed at age 40 and categorized based on the income quartiles for the working population, aged 20 to 65, in Sweden."

Are the analyses restricted to individuals who were working or restricted to this population when income is included in the model? Or do the authors mean that income was assessed for individuals in the age range 20 to 65, an age range where people tend to work? I would have liked that the authors commented on why they didn't include "working yes or no" or something related to employment as a covariate.
Response: We regret the lack of clarity. We did not restrict only to the working population, and have added that information to the Methods section (page 7, line 21). Employment status is

time-varying, and could change many times over the course of observation, making statistical modeling quite complicated. We hope that the Reviewer appreciates our perspective.

The authors write in the manuscript that income is "familial income", which I assume is the total income of the household, including both the index person and the partner? Please make this much clearer in the manuscript than what it is in the current. Related to the question above, does this mean that a person could be unemployed, but have an income in the 4th quartile if they have a partner that earns well? Please clarify.
Response: The reviewer is correct that an individual could be unemployed and still have an income in the 4th quartile if their partner earns well. We have clarified this in our description of the income and education variables in the Methods/Measures section: “These variables allow us to jointly account for, and distinguish the impact of, indices of individual-level SEP (educational level) and access to resources at the family level (familial income). An individual could have low educational attainment, and/or no personal income, but still have access to resources through a spouse’s income.”

Consider including some type of discussion regarding the use of familial income versus personal income as a measure of SEP. For example, the authors have chosen personal education level, not total educational level, so there must be a good reason for using household/familial income?
Response: We regret that our rationale for this approach was not adequately explained in our original submission. As noted in our response to the previous comment, we have now made clear that this allows us to account for both personal-level SEP and familial SEP (i.e., access to resources that one might not have otherwise).

Comment 2:
The authors’ highlights from the analyses results based on risk estimates of covariates. In other words, they start asking more research questions, such as: Does unmarried women have a higher risk of AMC then married women? Do Swede-Finns have a higher risk of AMC? These research questions or additional analyses should be mentioned earlier in the manuscript, as they are presented as important findings, but were not predefined.
The authors write that unmarried females were at reduced risk of AMC relative to married females. Now I am not an expert on causal pathways, DAGs, statistics and causality, but I have been told not to take a model specified to answer a causal research question (in this study, what is the association of SEP with AMC), and answer other research questions using the risk estimates from the covariates. If the model is set up for prediction (all variables in, then this is okey I was told).
I'm not saying it is wrong in this occasion, but the advice is probably to avoid ending up in situations where the model is not well suited to answer research questions pertaining to other variables than the exposure of interest. The other research question here is whether AMC risk differs by marital status, which is answered by a model set up to answer whether the risk of AMC differ by SEP. Please give it a thought, for example, consider whether adjusting the association between marital status and AMC risk for familial income is ok, as this is what is being done now I think. I guess it would place a lot of unmarried women without a partner in the "low income" section, and I don't know if that would be the best SEP indicator.
Response: We understand the Reviewer’s concern. While we do highlight a number of observations that were not key aspects of our original research question, we now clearly introduce these as “incidental findings” (page 17, line 8). We have dedicated considerably more of Discussion section to our pre-established research questions and interpretation of those findings, but felt it would be helpful for future studies to consider these “incidental” observations and design models accordingly. The text has been modified to read, “In addition to the effects of

our primary predictors of interest, we note three incidental findings that merit further investigation in the context of models specifically designed to replicate our observed associations.”

Comment 3:
Harmful and problematic alcohol consumption. Alcohol is defined as a Group 1 carcinogen (IARC). So, in that sense, any alcohol intake is harmful in a dose-dependent manner. How it interacts with mental health and emotions is well, difficult, bi-directional and perhaps multidirectional. I mean, alcohol is probably a part of celebrations as well as a bad coping mechanism.
The "not harmful" version stems from epidemiological studies of the traditional sort, showing that individuals reporting light to moderate, and in some cases also quite frequent intake, has a lower HR of ischemic heart disease in comparison with no/low/infrequent drinking. And because ischemic heart disease caused so many premature deaths among men in particular, and still does, but less so because of primary prevention (smoking and diet) and better treatment (medication and PCI), this association propagated into a lower risk of all-cause death in similar studies. Whether alcohol has a beneficial effect on ischemic heart disease is much more debated and controversial today, mainly because studies using instrumental variables does not find a beneficial association of alcohol with IHD (at least that was the case the last time I checked a couple of years ago), creating a discrepancy between results from "traditional" and "modern" observational studies. In the now published Nordic nutrition recommendations (revised every 8 years), "no alcohol intake" is promoted as the intake level that is associated with the lowest health risk of alcohol, taking into account alcohols effect on cancer, heart, as well as accidents and violence and drunken driving etc. I haven't found the background paper on this, but the conclusion seems to be based on the work with the Canadian low drinking guidelines, where the conclusion is as follows: "the most recent and highest quality systematic reviews showing that drinking a little alcohol neither decreases nor increases the risk of ischemic heart disease, but it is a risk factor for most other types of cardiovascular disease, including, hypertension, heart failure, high blood pressure, atrial fibrillation and hemorrhagic stroke"…
Paradis, C., Butt, P., Shield, K., Poole, N., Wells, S., Naimi, T., Sherk, A., & the Low-Risk Alcohol Drinking Guidelines Scientific Expert Panels. (2023). Canada's Guidance on Alcohol and Health: Final Report. Ottawa, Ont.: Canadian Centre on Substance Use and Addiction
Why am I writing this? The consensus might be moving in the direction that "any alcohol intake is harmful", which would be the case if there really is no beneficial effect of alcohol on the development of ischemic heart disease. Because the manuscript starts with referring to data from WHO regarding harmful alcohol use, and also use the word "problematic" alcohol use, which is in the same category, the authors states that one type of alcohol intake is harmful and indirectly that another type is not harmful.
I leave it up to the authors to decide whether they want to address this or not, but I encourage them to at least consider it. However, as a reader, I would prefer if the authors were more specific when they refer to literature regarding measures of alcohol intake and measures of health outcomes. When referring to intake as "harmful", what is the definition in the study? When referring to intake as "excessive", what is meant? When referring to a paper on heavy episodic drinking, what is the definition?
Response: This is a topic of ongoing concern, discussion, and debate within the alcohol research community, as the Reviewer very nicely illustrates. As the quality (and quantity) of data on alcohol intake and its association to a wide range of health outcomes improves, we might expect to continue seeing shifts in the empirical evidence for positive and negative effects of abstinence, low, and moderate drinking. Unfortunately, as the registry data does not include information on consumption level, in the current context we are unable to provide much insight

to this conversation. With respect to terminology (e.g., “harmful”, “excessive”), we have largely retained the original language as it reflects that used in the publications which we cite. However, the Reviewer makes an excellent point, and we have expanded our Limitations section to acknowledge points they raised and our inability to address them. The revised text reads (page 19, line 7): “Fourth, we were unable to directly account for differences in alcohol consumption, which is correlated with AUD, and which may differ by SEP. The medical community’s perspective on the impact of alcohol consumption at different levels is variable and subject to revision. For example, while many studies support a J-shaped association between consumption and certain health outcomes, other considerations (e.g., the climate-based impacts of the alcohol industry) have led some entities to recommend abstinence. These issues are beyond the scope of the current study but warrant further consideration for public health considerations.”

Comment 4:
Introduction, second paragraph: I think I understand what is meant with the term "lower occupational class", but mayhaps "it is better to write "lower socioeconomic position measured by occupation"?
Response: We understand the reviewer’s perspective here. We have elected to retain the original wording as it is consistent with that used in the paper we are citing.

Comment 5:
Although I understand the need to be short and there could be a word limit, consider whether "alcohol outcomes" is a term that is used extensively as an umbrella term for measures of alcohol intake and measures of health outcomes that is associated with different measures of alcohol intake.
Response: We understand the Reviewer’s point. When we first use the term “alcohol outcomes”, we provide examples of relevance for the current research questions. We believe this should orient the reader sufficiently.

Comment 6:
Individuals that died before the age of 40 years were not allowed to participate in this study, by design. It is perhaps worth mentioning and it should be commented on whether the authors think it is relevant for the association under study.
Response: The Reviewer makes a good point. In this revision, we now report the number of individuals who were excluded due to an AMC registration prior to age 40 as part of our provision of STROBE information. We selected age 40 based on the rarity of AMC registrations prior to that age, and to prioritize the relevance of income at age 40 and educational attainment (which is largely static by that point).

Comment 7:
Figure: Would it be correct to state that you have superimposed the hazard lines for each predictor?
Response: We agree that “superimposed” is preferrable to “collapsed” and have made this change.

Figure legend. There is no information in the figure legend that the hazard ratio is the risk of AMC. It now states that the hazard ratio is for education level and income.
There is no information on what is included in the models, so in order to make use of the figure, the reader must go back to method section. I have always included this information in the legends, but can't find anything about this in strobe guidelines or journal style that says what is

correct. You choose I guess. Might be that this part is not included in the pdf I have in front of me.
Response: We regret this oversight in our original submission. The figure legend now clearly states that the hazard ratios reflect the association between education/income (as the predictor[s] of interest) and risk of alcohol-related medical conditions. We also now indicate which covariates are included in each model.

Regarding the figures. At glance, I can't see any differences between the 4 figures for women or the 4 for men. If there is a difference, I need to use a ruler on the screen I think. The authors might consider if it is really necessary to include all 2x4 figures to show the same thing? If adding more variables to the equation did not materially alter HR for the predictors, then simply stating this in the text would be adequate for me at least. At least give it a thought.
Response: We can understand the Reviewer's perspective. We have elected to retain the original figures as we think it's relevant for readers to see how the effects of education/income are attenuated as we add more covariates across models.

Comment 8:
Supplemental tables: Wouldn't it be nice for the reader to have exact information on what is included in the model instead of having to twist and turn? There is no reason to not be super informative and helpful, given that the manuscript is being considered for an online journal, and this is supplemental information.
Response: We have expanded the tables to improve clarity. All variables included in each model are presented in the tables; in other words, there are no covariates for which the models have been adjusted for which we do not provide estimates.

Comment 9:
Result section:
Quoting: "We next estimated the association between lifetime educational attainment and income at age 40 in Models 1A and 1B, respectively"
This is not correct, is it? The outcome is AMC?
Response: We can see how our phrasing caused confusion. We have modified the text to read as follows (page 12, line 4): "We next estimated the association between lifetime educational attainment (in Model 1A) or income at age 40 (in Model 1B) with risk of AMC."

Quoting: "Among females (Figure and Supplementary Table 1), having the lowest level of education was initially associated with a nearly 5-fold increase in risk of AMC (HR=4.71; 95% CI 3.85, 5.77)."
Consider whether referring to the exact model is more precise than using "initially".
Response: Thank you for this suggestion, we have made this change and agree that it improves the clarity of the text.

Comment 10:
The reporting and use of interactions on an additive and multiplicative scale:
I am not a statistician and have to admit that I have had and still have problems wrapping my head around the use of reporting additive interactions as an alternative to multiplicative interaction:
The researchers ask the question of whether the association between education and income with the risk of AMC differs according to familial risk of AUD. Such a question is most often answered in a multiplicative model with interaction terms, which is what the researchers write that they have done.

If the interaction term shows a difference, this answers the research question. It is important to show the results of the test, and stratified HRs is often included to help the reader get an overview of how the nature of the interaction plays out for each group in comparison to each other, and in comparison with the results of the main model. That is what I expected to see, but the authors chose to report interaction on the additive scale from the multiplicative model. That is probably ok, but I have some minor comments.
Response: We have now added a reference to the Methods section (page 10, line 22) that helps explain our use of an additive interaction rather than a multiplicative interaction, and address the concerns below in turn.

Is it correct that results in table S11 are the same as S10, only with the interaction term? Seems to be materially the same, with slight variation.
Response: The Reviewer is correct that the only difference between the results in Supplementary Tables S10 and S11 is that the latter reflects a model that includes the interaction term. Because the interaction terms (RERI and S) are on the additive scale, we elected to relegate those estimates to a separate table (Supplementary Table S12).

The combination of what is presented in table S11 and S12 and the manuscript text, would you say that it provides the reader with a good understanding of how the association between income and AMC differs? The text explains it to me, and I can see from table S12 that the RERI and S looks different for education and income, but there is no guidance for me to understand the magnitude of the difference between the groups. That doesn't mean its wrong or anything, but perhaps help the reader a bit more in this regard.
Response: In our reading, the text provides the most helpful perspective on the results presented in Supplementary Tables S11 and S12, but we acknowledge that many readers process information in tabular format more readily. With respect to the magnitude in difference between the groups, we regret that we have not been able to identify a reference that helps readers effectively translate RERI/S estimates into effect sizes that could be considered “small”, “medium”, or “large” in the same way that, for example, Cohen’s d can be considered. RERI can range from negative to positive infinity (with 0 as the null), while S can range from 0 to positive infinity (with 1 as the null) – not especially informative, in our view. Thus, the most relevant perspective is whether the RERI is <0 or the S<1 (negative interaction), or whether the RERI is >0 or the S>1. We have added these details, which hopefully at least provide some additional context, to the Supplementary Methods section, with reference to Knol et al., 2011.

Knol MJ, VanderWeele TJ, Groenwold RH, Klungel OH, Rovers MM, Grobbee DE. Estimating measures of interaction on an additive scale for preventive exposures. Eur J Epidemiol. 2011;26(6):433-8. Epub 20110223. doi: 10.1007/s10654-011-9554-9. PubMed PMID: 21344323; PubMed Central PMCID: PMC3115067.

In addition, I agree with the authors that it is important not to make a big fuzz about small stuff. For example, if the group with familial AUD is for example very small, then it is not that important (from a public health perspective) that the association between low education or low income with the risk of AMC is more pronounced, in comparison with a scenario where this group is large. From a clinical point of view, and if it were treatment types or drugs involved, it would perhaps be clinical important for a practitioner to know about the interaction, even though the interaction involves only a very small group. As the authors includes this argument, I would really like to see how many that have a family risk of AUD calculated by the family matrix. Or maybe I missed it?
Response: We regret that this was not made clearer in our original submission. The FGRS is derived as a function of AUD registrations in each individual’s first- to fifth-degree relatives, and

is corrected for co-habitation in an effort to reduce the impact of the rearing environment and isolate genetic influences. The procedure has been described in detail in previous publications, and validated in different contexts. It is more closely akin to a family history density measure that has "covaried out" resemblance due to environmental factors than to a traditional family history measure. Because so many relatives are potentially included (as family size varies widely), and genetic relatedness is a central feature of the calculation – i.e., one first-degree family member with AUD "weighs" more than two fourth-degree family members – it is operationalized as a continuous variable. We standardize the variable so that it reflects a relative measure of aggregate genetic risk, so one unit corresponds to one standard deviation. We have slightly modified the FGRS description so that it reads as follows: "Finally, aggregate genetic liability for AUD, which was used in a series of secondary analyses, was operationalized using the family genetic risk score for AUD ($FGRS_{AUD}$), which assesses genetic risk by an examination of first through fifth degree relatives, correcting for cohabitation effects which could make relatives more similar in their AUD risk. This measure is a relative measure which is operationalized as a continuous, standardized indicator of aggregate genetic risk, so that one unit corresponds to one standard deviation in the population."
To respond to the Reviewer's question about "how many…have a family risk of AUD": As is the case for molecular polygenic scores, which the FGRS was created to approximate in genetically informative data but without genotypes, *everyone* does, just to a greater or lesser degree. We hope that our updated description of FGRS being a continuous, standardized variable makes this clearer.

Comment 11:
Quoting: "As noted above, previous research has found that drinking patterns vary by SEP, with those of lower SEP more likely to engage in heavy consumption (11, 12, 13, 14, 16), which is strongly linked to risk of AMC (22, 23, 24). Information on consumption levels is not available in the Swedish nationwide registries, precluding our ability to test whether the associations between education/income remain when accounting for heavy episodic drinking, for example. However, our finding that these associations persist when controlling for AUD - itself closely associated with consumption levels (25, 26) - is consistent with prior evidence that individuals of lower SEP are at disproportionately high risk of AMC even after accounting for heavy drinking (16)."
Heavy episodic drinking or binge drinking is the consumption of a lot of alcohol per occasion, normally defined as 5+ or 6+ units, corresponding to 60+ grams of pure alcohol per occasion. To qualify as HED, the person has to drink about 2 grams per day on average, which corresponds to 5+ units once a month. Heavy episodic drinking exist as a drinking pattern from this lower bound and up until the average daily consumption moves into the area of chronic heavy drinking or heavy drinking, which may be defined as >60 grams per day, which in practice means that the person can have a heavy episode drinking every day.
My point of writing this, is that the authors refers to heavy episodic drinking and heavy drinking/AUD without making a clear distinction about how they are defined. I also commented on this earlier with regards to the introduction. Chronic heavy drinking is probably much more strongly related to AMC, because the amount of alcohol is considerable and takes a big toll on the metabolizing organs of the body. You wouldn't expect the same thing for a person binge drinking once a week?
HED is very important in relation to alcohol-related accidents, and therefore also relevant for alcohol related mortality, but whether a given amount of alcohol consumed in the form of HED or spread over several days conveys a different effect on organs, and AMC, is not a research question that has not been studied extensively with good methods.
Perhaps better to only focus on AUD/heavy drinking/alcohol intake measured as the quantity, rather than mixing in HED in the discussion. Consider. I think maybe that this discussion

regarding alcohol intake should be made after the discussion that adjustment for AUD registrations did only slightly moderate the association of SEP with AMC. Could it be that the measurement of AUD is not very good a picking up alcohol intake that can lead to AMC? That is my interpretation. Any other interpretation would imply a link between SEP and AMC via another route than alcohol?
Response: We certainly appreciate the Reviewer's perspective on this point, and agree that distinctions between HED and AUD/intake are important to consider. In addition to making the aforementioned changes to our Limitations section, we have replaced "heavy episodic drinking" in the quoted Discussion section with "chronic heavy drinking".

Comment 12:
Quoting: "Third, we found that individuals with higher genetic liability to AUD were more susceptible to the adverse impact of low income, though this was not the case for those with lower levels of educational attainment. This could reflect the stronger overall effect size of income level, and replication in other samples, and perhaps using complementary methods (e.g., molecular polygenic scores) is needed."

Consider including the information that the test for interaction was performed at year 0, when the magnitude of the association of income with AMC was most pronounced.
Response: We appreciate this point and have modified the text accordingly (page 18, line 14): "This could reflect the stronger overall effect size of income level, and potentially due to the fact that the estimate is based on year 0, which is when the effect of income was most pronounced…"

Comment 13:
Quoting: "Finally, alcohol consumption varies across countries and cultures; the current findings might not generalize to other contexts."

I agree that this study is irrelevant for cultures where alcohol is not consumed, but this is obvious and does not leave the reader from another country with any meaningful insight about whether this study applies to their country. And alcohol consumption is not the only thing that can influence the external validity of these findings. A thorough discussion regarding the external validity of the findings is lacking in the current manuscript.

Take women for example, as the results are stratified by gender. They are likely to vary more between countries then men in terms of their alcohol intake, their educational attainment at age 40, and whether they work or not at age 40, for example depending on the access to kindergarten at a subsidized price. In Canada for example, the price for child care at day time is not the same as in Norway, which could contribute to more women choosing to stay home for more years. These are only thoughts.
Response: The Reviewer raises interesting and important questions about the generalizability of the study. We regret that, despite its many advantages, registry data from one country precludes our ability to evaluate how cultural differences in alcohol consumption, either overall or across sexes, relates to AMC risk (just to take the example above). While we are unable to address such issues here, we have added text to the Limitations section around this concern, touching on potential differences in alcohol accessibility and sexual egalitarianism that could impact associations between SEP and AMC risk.

Reviewer #3: This is a useful study based on a very large national cohort. The conclusions are well-supported by the data, and the strengths are many.
Response: We very much appreciate the Reviewer's positive feedback!

1) The authors refer to the outcome variable as "alcohol-related medical conditions". This is technically correct, but to add precision, I would recommend using the term "fully alcohol-attributable disorder or disease". As far as I could see from the list of diagnoses in the text, all values would qualify to be fully AADDs. That way, nobody would think that the article is about AUDs or broader health conditions that could be influenced by alcohol, such as cancer or stroke.
Response: We appreciate this suggestion. Because we have previously published a study using the Swedish registry data in which we used the term “alcohol-related medical conditions” (Edwards et al. 2021), we have elected to retain that terminology so that it is clear we are using the same construct.
Edwards AC, Sundquist K, Sundquist J, Kendler KS, Larsson Lonn S. Genetic and environmental influences on the progression from alcohol use disorder to alcohol-related medical conditions. Alcohol Clin Exp Res. 2021;45(12):2528-35. Epub 20211219. doi: 10.1111/acer.14731. PubMed PMID: 34923650; PubMed Central PMCID: PMC8712390.

2) In the abstract, the authors present the findings that are not based on genetic risk first, then the ones that are. However, the interaction with genetic risk is more cutting edge and has less uncertainty, so I would recommend that these are given focus in both the abstract and the title.
Response: We appreciate the Reviewer’s interest in our analyses that incorporate aggregate genetic liability. We have elected to retain the original ordering of the analyses for two reasons. First, we originally conceived of these analyses as accounting for region of origin in adjusted models, and want to remain faithful to that conception. Second, and perhaps more relevant, is that the genetically-informative analyses necessarily exclude individuals who did not have two Swedish born parents. We think that, while those analyses are interesting and important, they apply to a smaller population and therefore are less informative to our overall research question. Given the increasing importance of considering cultural context in substance use epidemiology, we have prioritized centering the analyses that include first- and second-generation immigrants.

3) The analysis treats death and emigration as similar to regular censoring. This will lead to biased estimates. The standard today is to use competing risks models, the type that was first developed by Fine and Gray (see Austin et al 2022 for an updated discussion). A competing risks regression model may not be estimable with such a large dataset, but perhaps the authors can run an analysis on a random sample of the population to estimate the influence of death. However, without considering death as a competing risk, the decline in risk associated with low education could be a complete artefact: it just seems that the risk declines over time, because those with low income and low education die disproportionately at a younger age, and are no longer at risk of AMD. I may be completely wrong here, but if so, I think the authors should explain why.
Response: We appreciate this suggestion. Our research team has some experience with competing risks models, and made an effort to construct such models to address the current research questions. However, due to the complicated nature of the variables (namely, the time-varying coefficients that are clearly important in the Cox models), this was ultimately not feasible. We take seriously the Reviewer’s concern that the decline in risk for education might be artifactual and therefore ran a sensitivity analysis where we tested a model that assumes all subjects censored at death, receive an AMC diagnosis at the time of death. The attenuations in association with education and subsequent AMC during follow-up were only slightly affected, so we concluded that the attenuation during follow-up is not an artifact.

4) The authors write that "The polychoric correlation between income quartile and education level for females was 0.185 (SE-0.001) and for males was 0.198 (SE=0.001), suggesting that

while these socioeconomic measures are related, they also provide non-overlapping information." This is not a very formal test of the degree of dependence or independence of the two variables. I think I get that the authors want to argue that both variables should be considered (and I agree), while they also want to acknowledge that both come under a common heading, SES (again - I agree). But if they want to provide a statistical argument for degree of dependence, I would want something stronger.
Response: We appreciate the Reviewer’s concern. We are not aware of an appropriate statistical test here: One can test for collinearity, but that would assume continuous variables and a linear relationship, neither of which is fulfilled. We have now included a cross-tabulation of income and education (Supplementary Tables S2-S3), and hope that helps assuage potential concerns.

Reference
Austin PC, Putter H, Lee DS, Steyerberg EW. Estimation of the Absolute Risk of Cardiovascular Disease and Other Events: Issues With the Use of Multiple Fine-Gray Subdistribution Hazard Models. Circ Cardiovasc Qual Outcomes. 2022.
