# Supplementary material for: Socioeconomic position indicators and risk of alcohol-related medical conditions: A national cohort study from Sweden
Source: PLoS Med. 2024 Mar 19;21(3):e1004359. doi: 10.1371/journal.pmed.1004359 (PMC10950249; doi:10.1371/journal.pmed.1004359)
Supplement: Attachment — Submitted filename: Responses to Editorial Comments.pdf [file pmed.1004359.s020.pdf]

Responses to Editorial Comments

COMMENTS FROM THE ACADEMIC EDITOR:

I agree with the decision to proceed.

I had just one more suggestion: Might it be helpful to clarify in the abstract that these results are from a high-income country? I would hypothesize that these risks would be very different in low-income countries. Sweden is a unique country in many ways.

Response: We are very grateful that our revision was well-received by the Editors and Reviewers. In the Limitations section of the Abstract, we have now noted that our results are limited to a high-income country.

COMMENTS FROM THE EDITORS:

GENERAL

Thank you for your detailed and considered responses to previous editor and reviewer comments. Please see below for further comments which we require you address prior to publication.

TITLE

Thank you for revising your title, we suggest, ‘Socioeconomic position indicators and risk of alcohol-related medical conditions: A national cohort study from Sweden.’

Response: We have made this change to the title.

ABSTRACT

Abstract methods and findings - ‘N=4253 (0.37%) of females and N=11,183 (0.93%) of males…’ please remove the ‘N=’ and the ‘of’ on both occasions here to improve reader accessibility.

Response: We have made the requested change to the abstract.

AUTHOR SUMMARY

Line 3 – suggest ‘…excess morbidity and mortality…’ instead.

Please add a final bullet point after line 24 of ‘What Do These Findings Mean?’, which briefly describes the main limitations of the study in non-technical language.

Response: We have made both suggested changes to the author summary.

INTRODUCTION

Page 6, line 5 – should the colon be a full stop?

Response: We think a colon is appropriate here, as the text following the colon explains what is meant by the text preceding the colon. However, we are happy to make the change if necessary.

METHODS and RESULTS

Results page 12 onwards - Multivariable models – if possible, we think that the presentation/description of your results could be made more concise to improve reader accessibility. It is not difficult to get a bit lost in the detail. Please revise for the purpose of improved brevity.

Response: We agree that the Results section was a bit unwieldy! We have streamlined it wherever possible to improve readability without detracting from what we believe are the most pertinent findings.
